# Stimulant Overdose Prediction Model for Medicaid-Insured Persons

**DOI:** 10.1001/jamahealthforum.2025.3489

**Published:** 2025-09-19

**Authors:** Tuhina Srivastava, Rebecca Arden Harris, Cheryl Bettigole, Hanxi Zhang, Colleen M. Brensinger, Kacie Bogar, Fengge Wang, Elizabeth D. Nesoff, Warren B. Bilker, Sean Hennessy

**Affiliations:** 1 Institute for Health Metrics and Evaluation, University of Washington, Seattle; 2 Department of Biostatistics, Epidemiology and Informatics, Perelman School of Medicine, University of Pennsylvania, Philadelphia; 3 Department of Family Medicine and Community Health, Perelman School of Medicine, University of Pennsylvania, Philadelphia; 4 Center for Clinical Epidemiology and Biostatistics, Perelman School of Medicine, University of Pennsylvania, Philadelphia; 5 Center for Real World Effectiveness and Safety of Therapeutics, Perelman School of Medicine, University of Pennsylvania, Philadelphia

## Abstract

**Importance:**

Overdoses involving methamphetamines and cocaine have increased in recent years. Identification of individuals at highest risk could facilitate the implementation of evidence-based interventions to reduce overdose risk.

**Objective:**

To develop and internally validate a model that predicts hospitalization or emergency department (ED) treatment for stimulant-involved overdose among the Medicaid-insured population.

**Design, Setting, and Participants:**

This was a retrospective case-cohort study using Medicaid claims data from 2016 to 2019 (development) and 2020 (validation) for all Medicaid enrollees age 15 years or older with a cocaine- or other stimulant−involved overdose. A subcohort was created using a simple random sample of the full cohort of all cases. Within the full cohort, cases were identified as those having any inpatient or ED encounter for stimulant-involved overdose during the following year. A case-cohort sample was obtained for each calendar year from 2016 to 2020, each with a subcohort size of 100 000. Each individual contributed only 1 case event (for an individual with multiple overdoses, only the first eligible was selected). For each of the 4 overdose outcomes, a predictive weighted Cox model was first developed among enrollees of sampling years 2016 to 2019 (development set), and its performance was evaluated in our test set of 2020. The prediction models were first developed in November 2023, and the model fairness assessment was performed in April to May 2025.

**Interventions or Exposures:**

Individual-level candidate predictors were demographic characteristics, enrollment, health care utilization, and other clinical variables. Area-level variables included social, economic, housing, and demographic characteristics data from the American Community Survey, rural-urban classification, Social Deprivation Index, retail opioid dispensing rates, and health resources.

**Main Outcomes and Measures:**

Four types of stimulant-involved overdose associated with hospitalization or ED treatment: cocaine-involved overdose, (1) involving an opioid or (2) not involving an opioid; or methamphetamine-, ecstasy-, or other psychostimulant-involved overdose (hereafter, other stimulant), (3) involving an opioid or (4) not involving an opioid.

**Results:**

The analysis included 78 795 enrollees with cocaine- and other stimulant−involved overdose (mean [SD] age, 42.2 [13.7] years; 33 304 [42%] female and 45 491 [58%] male individuals). Weighted Cox regression prediction models showed good calibration and high discriminatory performance (Harrell C statistic): cocaine-involved overdose, with (0.923) or without (0.902) an opioid; other stimulant−involved overdose, with (0.909) or without (0.868) an opioid. For cocaine-involved overdose with opioids, previous individual opioid use disorder diagnosis or cocaine use disorder diagnosis played the largest role in overdose risk prediction. For cocaine-involved overdose without opioids, previous cocaine use disorder diagnosis and area-level income inequality and housing variables contributed most to prediction. For other stimulant−involved overdose with opioids, previous opioid use disorder diagnosis and area-level percentage of those living with a disability contributed most to prediction. For other stimulant−involved overdoses without opioids, previous stimulant-related disorder and area-level proportion of individuals receiving Supplemental Nutrition Assistance Program contributed most to prediction.

**Conclusions and Relevance:**

This case-cohort study found that readily available data can be used to identify those at high risk of hospitalization or ED visit for cocaine- or stimulant-involved overdose. These individuals would likely benefit most from evidence-based interventions and awareness of risk factors for overdose.

## Key Points

**Question:**

Can a prediction model using individual- and area-level characteristics predict emergency department (ED) treatment and/or hospitalization for a stimulant-involved overdose ?

**Findings:**

This case-cohort study using Medicaid data for 78 795 Medicaid enrollees (2016-2020) found that a previous diagnosis of substance use disorder and certain area-level sociodemographic variables effectively predicted ED and/or hospital admission risk for overdose involving cocaine, methamphetamines, ecstasy, or other psychostimulants. The prediction models showed good calibration and excellent discrimination in predicting stimulant-involved overdose risk.

**Meaning:**

These findings indicate that prediction models using Medicaid and other available data can identify those at highest risk for cocaine- and methamphetamines-involved overdose, with or without opioid involvement.

## Teaser

This case-control study develops and validates a model to predict hospitalization or emergency department treatment for stimulant-involved overdose among the Medicaid-insured population.

## Introduction

The US drug overdose crisis remains a substantial public health problem, with 107 543 overdose deaths occurring in 2023.^1^ Overdose deaths that involve stimulants—cocaine or amphetamines—are frequently associated with myocardial infarction, arrhythmias, and cerebrovascular events triggered by excessive sympathetic nervous system activation. In the increasingly common situation in which the overdose death involves both stimulants and opioids, death may result from the effects of the opioid alone or from the combined effects of the drugs taken. Among drug overdose deaths in 2022, approximately 26% involved cocaine and 33% involved other psychostimulants with misuse potential such as methamphetamine.^1,2^ Drug overdose deaths involving methamphetamines increased 5-fold from 2015 to 2022, while overdose deaths involving cocaine increased 3-fold.^3^ The rate of nonfatal stimulant-involved overdose with or without the co-involvement of opioids resulting in inpatient or emergency department (ED) treatment has also increased.^4,5^

More than half of US amphetamine-related hospitalizations occur among Medicaid-insured persons.^6,7^ While evidence based approaches to reduce the risk of stimulant-involved overdose exist,^8^ there are currently no prediction models to aid in identifying those at highest risk across the US, although such models exist for opioid overdose.^9^ Variables that may be useful in identifying persons at high risk of stimulant-involved overdose include individual- and area-level measures of socioeconomic disadvantage, health services utilization indicators, and demographic variables.^7,10,11,12^ In addition to evidence-based messaging for the general public, those at greatest risk for stimulant-involved overdose identified by prediction models could be proactively offered evidence-based interventions. Public health practitioners could use these predictions to provide interventions such as community reinforcement or cognitive-behavioral therapy combined with contingency management—in which incentives are provided to reinforce healthy behaviors—as well as interventions that protect against the increasing risk from inadvertent or intentional intake of opioids in combination with stimulants, such as naloxone, fentanyl test strips, and guidance to avoid using alone.^13,14^

This study aimed to develop and internally validate a model to predict hospitalization or ED treatment for stimulant-involved overdose during the subsequent calendar year, using both individual- and area-level characteristics. Such a model would assist public health practitioners and policymakers to identify individuals at high risk of stimulant overdose, facilitating the delivery of evidence-based interventions and knowledge of potential risk factors for overdose to these individuals.

## Methods

This protocol was reviewed by the University of Pennsylvania Internal Review Board (Protocol No. 85003) and was deemed exempt, and a waiver of HIPAA (Health Insurance Portability and Accountability Act) authorization was granted because the Medicaid data claims used were deidentified and involved less than minimal risk to the privacy of the individuals. We followed the Preferred Reporting Items for Systematic Reviews and Meta-Analyses (PRISMA) reporting guideline.

### Data Sources and Study Population

Medicaid is a public insurance program in the US that provides health coverage to low-income families and individuals, including children, parents, pregnant persons, seniors, people with disabilities, and other income-eligible individuals.^15^ Given the large size of the population insured by Medicaid (80.9 million as of May 2024) and the relatively infrequent nature of stimulant-involved overdose, we used a case-cohort study design.^15,16^ This design uses data from all cases experiencing the outcome of interest, as well as a simple random sample of the full cohort referred to as the *subcohort*. The subcohort, together with all cases outside the subcohort, forms the case-cohort sample. In this study, the full cohort consisted of all Medicaid enrollees who were age 15 years or older at the beginning of a given calendar year. Within the full cohort, cases were identified as those having any inpatient or ED encounter for stimulant-involved overdose during the following year. Through the Virtual Research Data Center of the Centers for Medicare & Medicaid Services, a case-cohort sample was obtained for each calendar year, referred to as the *sampling year* hereafter, during 2016 to 2020, each with a subcohort size of 100 000.

Each individual contributed only 1 case event. If an individual had multiple eligible stimulant overdoses resulting in an ED encounter, the first eligible encounter was selected. Records were excluded if they (1) had any enrollment disruption during the year (referred to as the baseline year hereafter) immediately preceding the sampling year; (2) were cases outside the subcohort and had enrollment disruption before the first recorded stimulant-involved overdose during the sampling year; (3) showed place of residence as Puerto Rico, Virgin Islands, or missing data; or (4) had any area-level characteristic from the American Community Survey (ACS) missing. The rationale for these exclusion criteria was that a baseline period is needed to identify risk and protective factors, and because Medicaid programs seeking to identify and intervene for high-risk enrollees would need a baseline period during which to assess risk. Also, because Medicaid data formatting transitioned from the Medicaid Analytic eXtract (MAX) to the Transformed Medicaid Statistical Information System Analytic Files in 2015 for most states, which has many more data elements than MAX, records were excluded if the baseline year Medicaid data were in the MAX format. This criterion affected only sampling year 2016.

### Outcome and Predictor Variables

#### Outcome

The outcome was an inpatient or ED encounter indicating a stimulant-involved overdose in the sampling year and was identified based on relevant codes from the *International Statistical Classification of Diseases, Tenth Revision, Clinical Modification* in any position (ie, principal or nonprincipal) (eAppendix 1 in Supplement 1). We excluded poisoning by caffeine or methylphenidate, given that our focus was illicit stimulants and noninitial encounters (ie, follow-up visits).

Analyses were stratified by 4 different types of stimulant-involved overdose: (1) cocaine-involved overdose involving an opioid; (2) cocaine-involved overdose not involving an opioid; (3) methamphetamine-, ecstasy-, or other psychostimulant-involved (hereafter, other stimulant) overdose involving an opioid; and (4) other stimulant overdose not involving an opioid. Overdoses involving both cocaine and at least 1 of either methamphetamine, ecstasy, or other psychostimulant were included for both outcomes.

#### Predictor Variables

Candidate predictors consisted of individual- and area-level variables. eAppendix 2 in Supplement 1 provides the full list of variables and more details.

Individual-level variables included sociodemographic characteristics (eAppendix 2 in Supplement 1) collected from the Medicaid demographic and eligibility file, and baseline-year diagnoses and treatments (eg, behavioral and mental health, substance-related disorders, and prescription medications) derived from encounter data (eAppendix 2 in Supplement 1).

Area-level socioeconomic indicators were determined a priori based on a literature review identifying observed associations for stimulant overdose outcomes. These variables were sourced from the ACS (sampling year of Medicaid data corresponded to the last year of the 5-year data collection period), the Social Deprivation Index website, and Area Health Resource Files. Information regarding specific data years is available in eAppendix 2 in Supplement 1. We linked Medicaid individual-level data residence zip codes with Zip Code Tabulation Areas-level 5-year ACS estimates of area-level socioeconomic characteristics using a ZCTA-ZIP crosswalk.^17,18^ ACS variables included those related to income, education, employment, housing, household characteristics, transportation, and demographic characteristics (eg, age, race and ethnicity, sex).^18^ Race and ethnicity data were collected from the Transformed Medicaid Statistical Information System Analytic Files; other included Asian, American Indian or Alaska Native, Hawaiian or other Pacific Islander, and multiracial. We also included the following area-level socioeconomic inequality indicators: Gini Index,^19^ rural-urban classification,^20^ 2015 Social Deprivation Index scores,^21^ retail opioid dispensing rates,^22^ and health resources (eg, access to and availability of health care facilities, and clinicians).^23^

### Model Development and Validation

For each of the 4 overdose outcomes (types 1-4), a predictive model was first developed among enrollees of sampling years 2016 to 2019, which constituted the development set, and its performance was evaluated in our test set of 2020. Given our case-cohort design, we used weighted Cox models, with each individual weighted to account for the case-cohort sampling scheme.^16,24,25,26^ Barlow weights were used, which weight subcohort members by the inverse of their sampling probabilities to restore the full cohort.^25,27^ This type of weighting is useful when the sampling fraction (the ratio of the subcohort size over the full cohort size) is small.^28^ Each enrollee was followed up starting from January 1 of the sampling year, and ending at whichever came first, the overdose date, the Medicaid enrollment disruption date, or December 31 of the sampling year. Nonoverdose-related deaths were censored.

Because of the large number of candidate predictors and the infrequent nature of overdose events, we used penalized Cox regression to reduce overfitting and improve prediction accuracy.^29,30^ Specifically, weighted Cox regression with least absolute shrinkage selection operator (LASSO), stratified by sampling year, was performed in the development set, using the glmnet package for R.^31^ We performed variable selection using LASSO, a penalized regression method that shrinks the coefficients toward zero.^30,32^ The hyperparameter λ controlling the amount of penalty was selected using 10-fold cross-validation in the development set. The λ value producing the largest mean cross-validated Harrell C index was selected. Because the developed model was intended to be easily reused by Medicaid officials or others to predict stimulant-involved overdose risk, data preprocessing was kept to a minimum (eAppendix 2 in Supplement 1 provides additional information). A categorical variable was collapsed if the prevalence of at least 1 of its categories was less than 0.5% among cases for at least 1 outcome. The same candidate predictors were used for all 4 outcomes.

The discrimination and calibration of the developed models were subsequently assessed within the same development set (ie, apparent performance), and separately among the beneficiaries of sampling year 2020 (ie, an internal validation in the test set). Sensitivity and positive predictive values were calculated to assess model discriminatory performance for various predicted risk cutoffs (we have reported predicted risk of 5% to avoid small sample sizes and preserve confidentiality). Brier scores were calculated to determine the accuracy of the predictions. Calibration, or agreement between predicted and the observed stimulant-involved overdose risks at 1 year, was also assessed.^33^ Risk decile subgroups, as determined by the prognostic index, were used to assess model calibration. Performance metrics were calculated using the full development dataset. A TRIPOD checklist can be found in eAppendix 3 in Supplement 1. Model fairness assessments for sex and race and ethnicity variables showed no large differences between subgroups and can be found are in eAppendix 6 in Supplement 1. The prediction models were first developed in November 2023. The model fairness assessment was performed in April-May 2025.

## Results

The analyses included a total of 78 795 enrollees with cocaine- and other stimulant−involved overdose (mean [SD] age, 42.2 [13.7] years; 33 304 [42%] female and 45 491 [58%] male individuals) who experienced overdose from 2016 to 2020. The number of overdoses by type were cocaine-involved with opioid involvement (n = 8459); cocaine-involved without opioid involvement (n = 29 206); methamphetamine-, ecstasy-, or other psychostimulant-involved with opioid involvement (n = 6632); and methamphetamine-, ecstasy-, or other psychostimulant-involved without opioid involvement (n = 34 498) (Table 1; eAppendix 4 in Supplement 1). The individual- and area-level predictors selected using LASSO regression can be seen in Table 2 and eAppendix 5 in Supplement 1, along with their weighted Cox model hazard ratio results for each outcome. Tables 1 and 2 show the data for outcome for methamphetamine-, ecstasy-, or other psychostimulant-involved overdose without opioid involvement, while eAppendices 4 and 5 in Supplement 1, respectively, show the analogous data for the 3 other outcomes.

**Table 1.** Individual-Level Sociodemographic Characteristics of Medicaid Data on Other Stimulant−Involved Overdose Without Opioid Involvement^a^ Used for Prediction Model Development and Testing

Characteristic	No. (%)			
	Development (2016-2019)		Testing (2020)	
	Cases (n = 25 423)	Subcohort (n = 216 552)	Cases (n = 9075)	Subcohort (n = 62 004)
Sampling year				
2016	2533 (10.0)	26 465 (12.2)	2020 (100)	NA
2017	6857 (27.0)	62 929 (29.1)		
2018	7600 (29.9)	64 563 (29.8)		
2019	8433 (33.2)	62 595 (28.9)		
Age, mean (SD), y	38.2 (13.5)	41.6 (20.2)	39.6 (13.3)	42.1 (20.4)
Sex				
Female	10 967 (43.1)	128 236 (59.2)	3515 (38.7)	36 465 (58.8)
Male	14 456 (56.9)	88 316 (40.8)	5560 (61.3)	25 539 (41.2)
Race and ethnicity^b^				
Black (not Hispanic)	3024 (11.9)	39 838 (18.4)	1343 (14.8)	11 731 (18.9)
Hispanic (all races)	3825 (15.0)	39 572 (18.3)	1445 (15.9)	12 109 (19.5)
White (not Hispanic)	13 381 (52.6)	83 766 (38.7)	4800 (52.9)	24 844 (40.1)
Other	1344 (5.3)	15 706 (7.3)	528 (5.8)	4946 (8.0)
Missing	3849 (15.1)	37 670 (17.4)	959 (10.6)	8374 (13.5)
Household size, No. of people				
1	7088 (27.9)	46 782 (21.6)	2313 (25.5)	13 374 (21.6)
2	773 (3.0)	8842 (4.1)	255 (2.8)	2613 (4.2)
3	485 (1.9)	7067 (3.3)	172 (1.9)	2160 (3.5)
4	1027 (4.0)	13 766 (6.4)	317 (3.5)	4261 (6.9)
Missing	16 050 (63.1)	140 095 (64.7)	6018 (66.3)	39 596 (63.9)
Income relative to FPL				
0 to 100% of the FPL	7963 (31.3)	70 163 (32.4)	2678 (29.5)	20 961 (33.8)
≥101% of the FPL	557 (2.2)	15 701 (7.3)	197 (2.2)	4480 (7.2)
Missing	16 903 (66.5)	130 688 (60.3)	6200 (68.3)	36 563 (59.0)
Citizenship status				
Noncitizen	605 (2.4)	14 864 (6.9)	197 (2.2)	4446 (7.2)
US Citizen	20 667 (81.3)	154 076 (71.1)	7823 (86.2)	48 015 (77.4)
Missing	4151 (16.3)	47 612 (22.0)	1055 (11.6)	9543 (15.4)
HHS region				
Midwest	4296 (16.9)	49 404 (22.8)	1512 (16.7)	12 505 (20.2)
Northeast	1895 (7.5)	42 257 (19.5)	1002 (11.0)	14 320 (23.1)
Southeast	4397 (17.3)	41 609 (19.2)	1151 (12.7)	10 209 (16.5)
Southwest	2075 (8.2)	14 654 (6.8)	556 (6.1)	4117 (6.6)
West	12 760 (50.2)	68 628 (31.7)	4854 (53.5)	20 853 (33.6)
Dually eligible (Medicare and Medicaid), yes	2644 (10.4)	50 655 (23.4)	812 (8.9)	14 399 (23.2)
Comprehensive managed care plan, yes	20 437 (80.4)	161 791 (74.7)	7566 (83.4)	47 317 (76.3)
BHCMC plan, yes	3080 (12.1)	24 690 (11.4)	547 (6.0)	5194 (8.4)
Basis of Medicaid eligibility				
Child	2164 (8.5)	33 856 (15.6)	619 (6.8)	9699 (15.6)
Disability	4096 (16.1)	52 811 (24.4)	1244 (13.7)	13 759 (22.2)
Income	18 234 (71.7)	117 165 (54.1)	6963 (76.7)	34 941 (56.4)
Other	548 (2.2)	9869 (4.6)	187 (2.1)	2943 (4.7)
Missing	381 (1.5)	2851 (1.3)	62 (0.7)	662 (1.1)
Any disability, yes	6787 (26.7)	39 150 (18.1)	2243 (24.7)	10 557 (17.0)

Abbreviations: BHCMC, behavioral health comprehensive managed care; FPL, federal poverty level; HHS, US Department of Health and Human Services; NA, not applicable.

^a^ Type 4 overdose, ie, other stimulants including methamphetamines, ecstasy, and other psychostimulants without opioid involvement.

^b^ From the Transformed Medicaid Statistical Information System Analytic Files; other includes Asian, American Indian or Alaska Native, Hawaiian or other Pacific Islander, and multiracial.

^c^ Missing was included as a category where it was applicable in the individual-level data, to use the whole dataset, eg, for the race variable we had an indicator for missing vs non-Hispanic White [reference].

**Table 2.** Weighted Cox Model Hazard Ratio Results With LASSO-Selected^a^ Predictors for Other Stimulant−Involved^b^ Overdose Without Opioid Involvement Outcomes

Variable [reference group]	LASSO coefficient	HR (95% CI)	χ^2^ *P* value
Individual-level characteristics			
Age	−0.02	0.99 (0.98-0.99)	<.001
Sex: female [male]	−0.47	0.63 (0.61-0.65)	<.001
Race: Black [White, not Hispanic]	−0.31	0.74 (0.70-0.77)	<.001
Race: other [White, not Hispanic]	−0.14	0.87 (0.82-0.92)	<.001
Race: Hispanic [White, not Hispanic]	−0.24	0.79 (0.76-0.82)	<.001
Race: Missing [White, not Hispanic]	−0.16	0.85 (0.82-0.89)	<.001
US region: Midwest [West]	−0.81	0.44 (0.42-0.47)	<.001
US region: Northeast [West]	−1.15	0.32 (0.30-0.34)	<.001
US region: Southeast [West]	−0.43	0.65 (0.62-0.68)	<.001
Household size: 3 people [1 person]	−0.21	0.81 (0.74-0.89)	<.001
Household size: ≥4 people [1 person]	−0.07	0.94 (0.87-1.00)	.06
Household size: missing [1 person]	0.03	1.03 (0.99-1.07)	.12
Immigration status: qualified noncitizen [US citizen]	−0.43	0.65 (0.55-0.77)	<.001
Income relative to FPL: ≥101% [0%-100%]	−0.47	0.63 (0.58-0.68)	<.001
Marital status: married [never married/partnered]	−0.37	0.69 (0.63-0.77)	<.001
Primary language spoken: other [English]	−1.20	0.30 (0.27-0.33)	<.001
Primary language spoken: missing [English]	−0.30	0.74 (0.71-0.77)	<.001
TANF benefits: did receive benefits [did not receive TANF]	−0.29	0.75 (0.71-0.79)	<.001
TANF: missing [did not receive TANF]	0.27	1.31 (1.26-1.36)	<.001
Patient dually eligible for Medicaid and Medicare	−0.48	0.62 (0.59-0.65)	<.001
Basis of Medicaid eligibility: child [income]	−0.51	0.60 (0.57-0.64)	<.001
Basis of Medicaid eligibility: disability [income]	−0.26	0.77 (0.74-0.80)	<.001
Citizenship status: noncitizen [US citizen]	−0.36	0.70 (0.61-0.80)	<.001
Area-level characteristics			
Gini index	1.25	3.48 (2.46-4.92)	<.001
% Population <100% FPL	0.83	2.30 (1.67-3.19)	<.001
% Total households receiving food stamps/SNAP in the past 12 mo	−2.87	0.06 (0.04-0.08)	<.001
% Population ≥25 y and with <12 y of education	1.66	5.25 (3.97-6.93)	<.001
% Population living in crowded housing units	2.88	17.89 (11.91-26.86)	<.001
Proportion of renter-households that are estimated to spend ≥35% of income on rent	0.51	1.67 (1.43-1.96)	<.001
% Vacant homes	−0.95	0.39 (0.31-0.48)	<.001
% Households with female heads and children <18 y	0.41	1.51 (1.09-2.10)	.01
% Workers who drive alone with a commute >30 min	−0.16	0.85 (0.76-0.95)	.003
% Population with high need (age <5 y plus women 15-44 y plus all ≥65 y)	−0.93	0.39 (0.23-0.67)	<.001
% Male	1.36	3.90 (2.13-7.16)	<.001
% Hispanic White [White, not Hispanic]	0.69	1.99 (1.80-2.20)	<.001
% Living with a disability	2.86	17.44 (11.56-26.29)	<.001
% Total population remaining in the same residence for the past 5 y	−0.06	0.94 (0.77-1.15)	.57
% Population <65 y without health insurance	−1.22	0.30 (0.21-0.41)	<.001
Employment rate of workforce employed in manufacturing jobs per 1000 residents	0	1.00 (1.00-1.00)	<.001
Employment rate of workforce employed in professional/service jobs per 1000 residents	0	1.00 (1.00-1.00)	<.001
Median household income in the past 12 mo	0	1.00 (1.00-1.00)	<.001
Retail opioid prescriptions dispensed per 100 persons per year	0	1.00 (1.00-1.00)	.43
Total specialists per 100 000 population; available years, 2015 and 2018	0	1.00 (1.00-1.00)	.37
Total hospitals per 100 000 population	0.01	1.01 (1.00-1.02)	.01
Degree of urbanization and adjacency to a metro area (2010 version): large rural [urban]	0.11	1.12 (1.07-1.17)	<.001
Indicator for mental care shortage: whole [part of county designated as shortage area]	0.08	1.08 (1.05-1.12)	<.001
Individual-level clinical characteristics			
Antibiotics	0.01	1.01 (0.98-1.05)	.38
Antidepressants	0.00	1.00 (0.96-1.03)	.91
Antipsychotics	0.43	1.53 (1.47-1.59)	<.001
Anxiolytics	0.16	1.17 (1.13-1.21)	<.001
Buprenorphine	−0.11	0.89 (0.84-0.95)	<.001
Gabapentinoids	0.18	1.19 (1.15-1.24)	<.001
Treatments for hepatitis C	−0.60	0.55 (0.46-0.66)	<.001
Methadone	0.13	1.14 (1.05-1.23)	.002
Mood stabilizing agents	−0.19	0.83 (0.79-0.87)	<.001
Naltrexone	−0.17	0.84 (0.77-0.92)	<.001
Opioids	0.05	1.06 (1.02-1.09)	<.001
Stimulants	0.63	1.88 (1.78-1.98)	<.001
ED visits in baseline year, No.	0.02	1.02 (1.02-1.02)	<.001
Was patient in BHCMC plan during baseline year?	−0.19	0.83 (0.79-0.87)	<.001
Individual-level clinical diagnoses			
Abscess or cellulitis: any position OT/LT diagnosis during baseline year	0.25	1.28 (1.23-1.33)	<.001
ADHD: any position IP diagnosis during baseline year	1.08	2.94 (2.68-3.22)	<.001
ADHD: any position OT/LT diagnosis during baseline year	0.32	1.37 (1.30-1.46)	<.001
Alcohol disorder: any position OT/LT diagnosis during baseline year	0.62	1.87 (1.79-1.95)	<.001
Alcohol disorder: nonprimary IP diagnosis during baseline year	0.55	1.73 (1.63-1.83)	<.001
Anxiety: any position IP diagnosis during baseline year	0.03	1.03 (0.98-1.09)	.25
Anxiety: any position OT/LT diagnosis during baseline year	0.23	1.25 (1.21-1.30)	<.001
Asthma: any position IP diagnosis during baseline year	−0.16	0.85 (0.79-0.91)	<.001
Asthma: any position OT/LT diagnosis during baseline year	−0.13	0.88 (0.83-0.92)	<.001
Bipolar disorder: any position OT/LT diagnosis during baseline year	0.37	1.45 (1.39-1.51)	<.001
Bipolar disorder: primary IP diagnosis during baseline year	−0.24	0.79 (0.72-0.86)	<.001
Cannabis: any position IP diagnosis during baseline year	−0.13	0.88 (0.82-0.95)	<.001
Cannabis: any position OT/LT diagnosis during baseline year	0.18	1.20 (1.13-1.27)	<.001
Cardiovascular: any position IP diagnosis during baseline year	0.84	2.32 (2.19-2.46)	<.001
Cardiovascular: any position OT/LT diagnosis during baseline year	0.33	1.39 (1.31-1.47)	<.001
Chronic kidney disease: any position IP diagnosis during baseline year	0.53	1.70 (1.61-1.79)	<.001
Chronic kidney disease: any position OT/LT diagnosis during baseline year	0.15	1.16 (1.10-1.23)	<.001
Cocaine: any position IP diagnosis during baseline year	−0.38	0.69 (0.63-0.75)	<.001
Cocaine: any position OT/LT diagnosis during baseline year	0.28	1.33 (1.23-1.43)	<.001
Depression disorder: any position OT/LT diagnosis during baseline year	0.20	1.22 (1.18-1.27)	<.001
Depression disorder: nonprimary IP diagnosis during baseline year	0.23	1.26 (1.19-1.34)	<.001
Depression disorder: primary IP diagnosis during baseline year	0.31	1.36 (1.26-1.47)	<.001
Hallucinogen-related disorders: any position IP, OT, or LT diagnosis during baseline year	1.09	2.96 (2.55-3.44)	<.001
Hepatitis C: any position IP diagnosis during baseline year	0.29	1.34 (1.22-1.47)	<.001
Hepatitis C: any position OT/LT diagnosis during baseline year	0.37	1.45 (1.35-1.55)	<.001
HIV: any position IP, OT, or LT diagnosis during baseline year	0.68	1.97 (1.81-2.13)	<.001
Hypertension: any position IP diagnosis during baseline year	−0.18	0.83 (0.79-0.88)	<.001
Infectious endocarditis: any position IP, OT, or LT diagnosis during baseline year	0.02	1.02 (0.89-1.17)	.81
Inhalant-related disorders: any position IP, OT, or LT diagnosis during baseline year	0.60	1.83 (1.57-2.13)	<.001
Lung disease: any position OT/LT diagnosis during baseline year	0.19	1.20 (1.15-1.26)	<.001
Manic episode: any position IP diagnosis during baseline year	−1.08	0.34 (0.29-0.40)	<.001
Manic episode: any position OT/LT diagnosis during baseline year	0.28	1.32 (1.22-1.42)	<.001
Nicotine: any position IP diagnosis during baseline year	0.64	1.90 (1.81-1.99)	<.001
Nicotine: any position OT/LT diagnosis during baseline year	0.50	1.65 (1.58-1.71)	<.001
OUD: any position OT/LT diagnosis during baseline year	0.36	1.43 (1.36-1.50)	<.001
OUD: nonprimary IP diagnosis during baseline year	−0.38	0.69 (0.64-0.74)	<.001
OUD: primary IP diagnosis during baseline year	0.16	1.17 (1.02-1.34)	.03
Other psychoactive substance: any position IP diagnosis during baseline year	0.99	2.70 (2.51-2.90)	<.001
Other psychoactive substance: any position OT/LT diagnosis during baseline year	0.47	1.59 (1.52-1.67)	<.001
Personality disorders: any position IP diagnosis during baseline year	0.31	1.37 (1.24-1.50)	<.001
PTSD: any position IP diagnosis during baseline year	−0.10	0.91 (0.84-0.99)	.02
PTSD: any position OT/LT diagnosis during baseline year	0.16	1.18 (1.12-1.24)	<.001
Schizophrenia: any position OT/LT diagnosis during baseline year	0.29	1.33 (1.28-1.39)	<.001
Schizophrenia: nonprimary IP diagnosis during baseline year	0.23	1.26 (1.16-1.37)	<.001
Schizophrenia: primary IP diagnosis during baseline year	0.29	1.33 (1.23-1.45)	<.001
Sedative/hypnotics/anxiolytic: any position IP diagnosis during baseline year	−0.01	0.99 (0.88-1.11)	.85
Sedative/hypnotics/anxiolytic: any position OT/LT diagnosis during baseline year	0.11	1.11 (1.01-1.23)	.04
Sexually transmitted infections: any position IP diagnosis during baseline year	0.27	1.31 (1.09-1.57)	.004
Sexually transmitted infections: any position OT/LT diagnosis during baseline year	0.24	1.27 (1.19-1.37)	<.001
Sleep disorders: any position OT/LT diagnosis during baseline year	−0.19	0.83 (0.79-0.87)	<.001
Stimulants: any position IP diagnosis during baseline year	2.22	9.23 (8.73-9.75)	<.001
Stimulants: any position OT/LT diagnosis during baseline year	1.80	6.08 (5.82-6.36)	<.001

Abbreviations: ADHD, attention-deficit hyperactivity disorder; BHCMC, behavioral health comprehensive managed care; ED, emergency department; FPL, federal poverty line; HR, hazard ratio; IP, inpatient; LASSO, least absolute shrinkage selection operator; LT, long-term care; OT, other services; OUD, opioid use disorder; PTSD, posttraumatic stress disorder; SNAP, Supplemental Nutrition Assistance Program; TANF, Temporary Assistance for Needy Families.

^a^ LASSO parameter estimates that show 0.00 are nonzero values that have been included in the model. 0.00 indicates that the value is small enough to be rounded to 0.00 when using 2 digits after the decimal.

^b^ Type 4 overdose, ie, other stimulants including methamphetamines, ecstasy, and other psychostimulants without opioid involvement.

^c^ Variables regarding pharmaceuticals (listed under individual-level clinical characteristics) during the calendar year preceding the sampling year.

^d^ α = .05.

^e^ Results for the outcomes: type 1 overdose, cocaine-involved with opioid involvement; 2, cocaine-involved without opioid involvement; and 3, methamphetamine-, ecstasy-, or other psychostimulant−involved with opioid involvement are reported in eAppendix 5 in Supplement 1.

### Predictive Model Performance

Table 3 shows the distribution and proportion of predictions of overdose made by predicted risk decile for each outcome, with the higher numbered deciles indicating those individuals with higher overdose risk. For each prediction model, over 75% of predicted overdose cases fell in the highest 2 risk deciles (Table 3).

**Table 3.** Frequency and Distribution of Cases, by Deciles of Predicted Risk of Outcome in the Test Set

Risk decile	No. (%)									
	Lowest risk		3	4	5	6	7	8	Highest risk	
	1	2							9	10
**Testing model, 2020**										
(1) Cocaine-involved with opioid involvement (n = 1912)	<11	<11	21 (1.1)	23 (1.2)	34 (1.8)	37 (1.9)	63 (3.3)	79 (4.1)	162 (8.5)	1483 (77.6)
(2) Cocaine-involved without opioid involvement (n = 6746)	31 (0.5)	55 (0.8)	74 (1.1)	100 (1.5)	124 (1.8)	155 (2.3)	245 (3.6)	389 (5.8)	702 (10.4)	4871 (72.2)
(3) Methamphetamine-, ecstasy-, or other psychostimulant−involved with opioid involvement (n = 1724)	<11	<11	<11	21 (1.2)	24 (1.4)	52 (3.0)	66 (3.8)	116 (6.7)	154 (8.9)	1274 (73.9)
(4) Methamphetamine-, ecstasy-, or other psychostimulant−involved without opioid involvement (n = 9075)	29 (0.3)	64 (0.7)	104 (1.1)	194 (2.1)	260 (2.9)	357 (3.9)	456 (5.0)	711 (7.8)	1075 (11.8)	5825 (64.2)

#### Type 1−Cocaine-Involved Overdose With Opioid Involvement

This model had 136 covariates as selected by LASSO (eAppendix 5 in Supplement 1). The median (IQR) time to overdose in the development cohort was 173 (90-257) days. The Brier score, which measures the accuracy of a set of probabilistic predictions, for this model was 0.031, indicating excellent accuracy (Table 4). The Harrell C statistic for this model was 0.923, indicating excellent discriminative performance. The 5 variables that contributed most to the model were primary inpatient opioid use disorder (OUD) diagnosis; nonprimary inpatient OUD diagnosis; OUD diagnosis in a long-term care (LTC) facility and those receiving other services; inpatient cocaine-related diagnosis; and cocaine-related diagnosis in LTC facilities and those receiving other services. The mean calibration ratio, also known as the *calibration-in-large* using the ratio of Kaplan-Meier event rate to average predicted risk to measure agreement of the predicted and observed, was 0.83 (95% CI, 0.74-0.94), indicating some underestimation of overdose risk. The calibration plot (Figure) demonstrated strong predictive performance, suggesting good calibration but with slight overestimation at lower prognostic indices (low risk of overdose) and some underestimation at higher prognostic indices (high risk of overdose).

**Table 4.** Predictive Modeling Performance on Test Set (2020) Using Brier Score by Outcome and Sensitivity and Positive Predictive Value (PPV) With a Predicted Risk of 5% or Greater Outcome Threshold

Model outcome	Brier score	Predicted risk ≥5%, %	
		Sensitivity	PPV
(1) Cocaine with opioid involvement	0.031	1	5
(2) Cocaine only without opioid involvement	0.098	12	7
(3) Other stimulant with opioid involvement	0.028	1	4
(4) Other stimulant without opioid involvement	0.129	7	6

**Figure.**
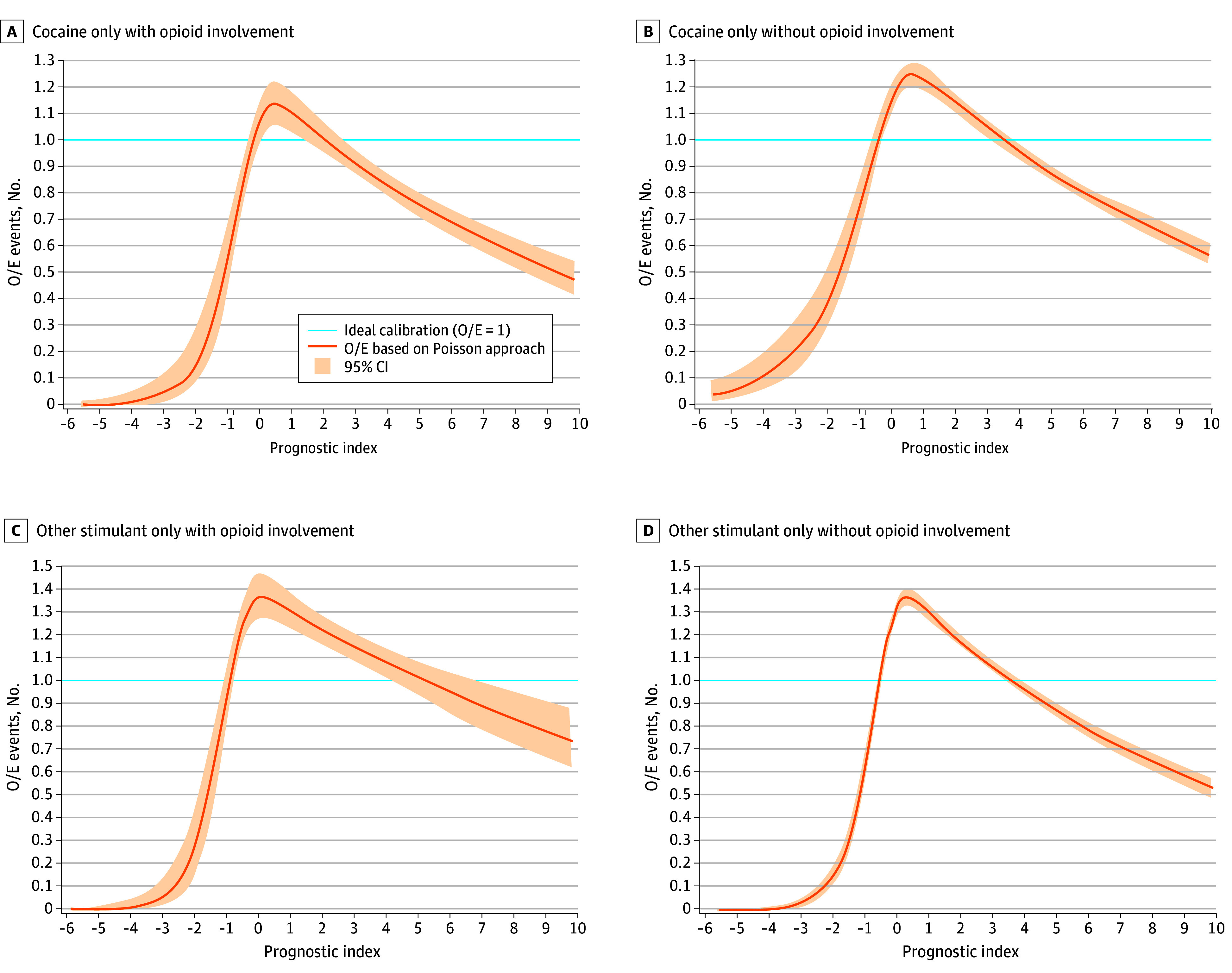
Calibration Plot of Observed (O) vs Expected (E) Outcomes for the Weighted Cox Regression Model in the Test Set, 2020 The prognostic index represents a composite risk score indicating the predicted likelihood of the outcome occurring. A higher value indicates a greater relative risk of the outcome, while lower (negative) values reflect lower relative risk. It is calculated as the weighted sum of the model’s covariates multiplied by their estimated coefficients, and as is standard with Cox models, the prognostic index is unbounded and can take both negative and positive values, depending on the combination of covariate values. The observed patterns in the calibration plot—where higher prognostic index values appear to correspond to lower observed event rates—are likely due to small sample sizes or instability in the extreme tails of the risk distribution. The y-axis shows the ratio of observed events to expected events. A ratio of 1 represents perfect calibration, meaning the model’s predictions match the observed event rates. Ratios >1 indicate that more events occurred than expected, while ratios <1 suggest that the model is underestimating the number of events.

#### Type 2−Cocaine-Involved Overdose Without Opioid Involvement

This model had 153 covariates as selected by LASSO (eAppendix 5 in Supplement 1). The median (IQR) time to overdose in the development cohort was 173 (87-263) days. The Brier score for this model was 0.098, indicating reasonable accuracy (Table 4). The Harrell C statistic for this model was 0.902, indicating excellent discriminative performance. The 5 variables that contributed most to the model were inpatient cocaine-related diagnosis, Gini index (area-level summary measure of income inequality), cocaine-related diagnosis in LTC facilities and those receiving other services, area-level percentage of individuals 16 to 64 years of age that were unemployed, and area-level proportion of renter households that are estimated to spend at least 35% of their income on rent. The mean calibration ratio was 0.95 (95% CI, 0.85-1.07), indicating only a slight underestimation of overdose risk. The calibration plot (Figure) demonstrated strong predictive performance, suggesting good calibration but with some overestimation at lower prognostic indices and slight underestimation at higher prognostic indices.

#### Type 3−Other Stimulant−Involved Overdose With Opioid Involvement

This model had 124 covariates as selected by LASSO (eAppendix 5 in Supplement 1). The median (IQR) time to overdose in the development cohort was 175 (89-258) days. The Brier score for this model was 0.028, indicating excellent accuracy (Table 4). The Harrell C statistic was 0.909, indicating excellent discriminative performance for the model. The 5 variables that contributed most to the model include: area-level percentage male, area-level percentage living with a disability, primary inpatient OUD diagnosis, nonprimary inpatient OUD diagnosis, and OUD diagnosis in LTC facility and those receiving other services. The mean calibration ratio was 1.13 (95% CI, 1.01-1.27), indicating some overestimation of overdose risk. The calibration plot (Figure) demonstrated good alignment between predicted and observed event rates for most prognostic indices, with some overestimation at lower prognostic indices and slight underestimation at higher prognostic indices.

#### Type 4−Other Stimulant−Involved Overdose Without Opioid Involvement

The Brier score for this model was 0.129, indicating reasonable accuracy (Table 4). The Harrell C statistic for this model was 0.868, indicating strong discriminative performance. The 5 variables that contributed most to the model included: area-level percentage population living in crowded housing units, area-level percentage living with a disability, inpatient stimulant-related disorder diagnosis, stimulant-related disorder diagnosis in LTC facility and those receiving other services, and area-level percentage receiving Supplemental Nutrition Assistance Program. The mean calibration ratio was 1.03 (95% CI, 0.92-1.16), indicating good calibration with only a slight overestimation of risk. The calibration plot (Figure) demonstrated strong predictive performance, with the observed-to-expected event ratio staying close to 1 across most prognostic index values, suggesting good calibration but with some overestimation at lower prognostic indices and some underestimation at higher prognostic indices.

## Discussion

We used Medicaid claims data from 2016 to 2020 and other publicly available data to predict next-year hospitalization or ED treatment for stimulant-involved overdose, a critical public health problem in the US. Overall, the prediction models performed well and were able to predict which patients would be at highest risk (categorized by risk decile or absolute-risk threshold) for each of the outcomes, indicating their potential usefulness in identifying those persons who may benefit most from evidence-based interventions. Furthermore, area-level indicators that played a substantial role in overdose risk prediction could be used to prioritize zip codes for scarce resource allocation for these interventions.

For cocaine-involved overdose with opioid involvement, variables documenting previous OUD or CUD had the highest contribution to the model. The second highest number of cases (n = 29 206) in this dataset came from cocaine-involved overdoses without opioid involvement. For this outcome, variables documenting previous OUD or CUD diagnoses had the highest contribution to the model. While variable indicating previously documented CUD diagnosis contributed to the model, zip code−level indicators of higher income inequality (Gini index), unemployment, and high rent played substantial roles in predicting risk. These results underscore the importance of understanding the interplay between individual-level and area-level sociodemographic factors on cocaine overdose risk. While these analyses were intended for prediction rather than to inform causal inference, the importance of these zip code−level indicators suggest that social disconnection and stress stemming from income inequality and housing instability due to high rent may contribute to overdose risk, as is previously indicated in the literature.^12,34^

Other stimulant−involved overdose risk with opioid involvement, variables documenting previous OUD diagnosis had the largest contributions to the model. This suggests that individuals who already have OUD documented in their claims history may be most vulnerable to overdose, and therefore, the best candidates to target for interventions. This diagnosis coupled with zip code−level indicators of areas with higher percentage male populations and individuals living with disabilities played the largest role in predicting other stimulant−involved overdose risk with opioid involvement. The highest number of cases (n = 34 498) in this study came from other stimulant−involved overdoses without opioid involvement. Previous stimulant-related disorder diagnosis had the highest contribution to the model predicting this specific outcome. Diagnoses of stimulant-related disorders, coupled with zip code−level indicators of areas with higher percentage of the population living in crowded housing units and individuals living with disabilities, played the largest role in predicting risk of other stimulant−involved overdose without opioid involvement. Housing instability has been shown to exacerbate overdose-related mortality in more resource-deprived areas, highlighting the intersectional nature of structural inequalities and stimulant-involved overdose.^35^ Furthermore, the presence of a disability at the individual level has also been associated with overdose risk, further supporting our study findings.^35,36,37^

The derived predictive models had excellent performance based on the Brier score for all 4 outcomes (type 1-4). They identified individuals at elevated risk for overdose; however, sensitivity and positive predictive value (PPV) were modest but expected because this is a well-documented challenge in predicting rare outcomes.^38^ PPV is mathematically constrained for rare outcomes, even with strong model calibration and discrimination.^38^ Sensitivity may be reduced when using a conservative threshold (eg, ≥5% predicted risk) to define high-risk individuals. This threshold favors specificity and PPV, but can miss true cases, leading to lower sensitivity. However, this trade-off is justifiable when interventions carry cost or resource constraint, thus prioritizing identification of those at highest-risk of overdose, rather than all potential cases. Importantly, despite lower sensitivity and PPV, our models retain value as a risk stratification tool to supporting public health decisions by flagging individuals for further preventive strategies or tailored intervention. Furthermore, the mean calibration ratios for our models were almost all close to 1 with narrow confidence intervals, indicating reasonable calibration; that is, how well the model’s predicted risks agree with observed outcome frequencies. The predicted risk deciles and thresholds that were reported in this study were chosen simply to demonstrate how these models could be evaluated. Depending on whether underestimation or overestimation is more acceptable from a policy perspective, these models could be calibrated to modify their performance.

### Strengths and Limitations

Although our models included many more variables than what may traditionally be considered parsimonious, our goal was to create a predictive model with the best predictive performance, rather than estimate the causal effect of each predictor. Furthermore, if such predictive models are used by Medicaid programs, the pragmatic and most useful approach may be to include all available data to make the best predictions. Given these considerations, we performed LASSO regression to select variables and did not further preprocess the data to limit the number of predictors. A strength of this approach is to make the modeling approach easily reproducible and usable for large datasets. Furthermore, using this Medicaid dataset, which is a large and diverse population rather than that of a single or multiple local health systems, makes these results more broadly generalizable.

Another limitation is that the model predicts only the first overdose event, whereas the literature suggests that prior overdose history is a strong predictor of recurrence. This is a potential future direction for these analyses. In this work, we also did not account for fatal overdoses that did not result in health care (which are few of the fatal overdoses) and did not link to death certificate data. This may have allowed an underestimation of cases by our model. However, because our goal was to create a novel prediction model that was easily replicable by Medicaid officials and other policymakers, linking of death certificate data would greatly complicate the adoption of these methods for what we hypothesize would be a marginal benefit.

## Conclusions

This case-cohort study found that Medicaid and other readily available data are potentially useful for predicting which individuals are most vulnerable to stimulant-involved overdose. The largest number of overdose cases were of methamphetamines, ecstasy, or other psychostimulant or cocaine-involved overdose, without opioid involvement. Individual-level variables related to previous diagnosis with specific substance use disorders, as well as key sociodemographic zip code−level variables indicating more vulnerable communities, contributed the most to the predictions made by these models. Our findings show that novel prediction models perform well and may be useful in identifying individuals who may benefit most from resource-intensive evidence-based interventions to prevent stimulant-involved overdoses.

## Notes

Supplement 1. eAppendix 1. Patient identification and ICD-10-CM inclusion codes by stimulant-involved overdose outcome eAppendix 2. Detailed information about the study methods eAppendix 3. TRIPOD Checklist: Prediction Model Development eAppendix 4. Additional individual-level sociodemographic characteristics of Medicaid claims data (2016-2020) used for development and testing by outcome eAppendix 5. Additional Weighted Cox model hazard ratio results with LASSO-selected predictors by outcome eAppendix 6. Model fairness assessment with regards to sex and race/ethnicity variables

Supplement 2. Data Sharing Statement
