# Supplementary material for: Stimulant Overdose Prediction Model for Medicaid-Insured Persons
Source: JAMA Health Forum. 2025 Sep 19;6(9):e253489. doi: 10.1001/jamahealthforum.2025.3489 (PMC12449722; doi:10.1001/jamahealthforum.2025.3489)
Supplement: Supplement 1. — eAppendix 1. Patient identification and ICD-10-CM inclusion codes by stimulant-involved overdose outcome eAppendix 2. Detailed information about the study methods eAppendix 3. TRIPOD Checklist: Prediction Model Development eAppendix 4. Additional individual-level sociodemographic characteristics of Medicaid claims data (2016-2020) used for development and testing by outcome eAppendix 5. Additional Weighted Cox model hazard ratio results with LASSO-selected predictors by outcome eAppendix 6. Model fairness assessment with regards to sex and race/ethnicity variables [file jamahealthforum-e253489-s001.pdf]

Supplemental Online Content

Srivastava T, Harris RA, Bettigole C, et al. Stimulant overdose prediction model for Medicaid-insured persons. *JAMA Health Forum.* Published online September 19, 2025. doi:10.1001/jamahealthforum.2025.3489

eAppendix 1. Patient identification and ICD-10-CM inclusion codes by stimulant-involved overdose outcome

eAppendix 2. Detailed information about the study methods

eAppendix 3. TRIPOD Checklist: Prediction Model Development

eAppendix 4. Additional individual-level sociodemographic characteristics of Medicaid claims data (2016-2020) used for development and testing by outcome

eAppendix 5. Additional Weighted Cox model hazard ratio results with LASSO-selected predictors by outcome

eAppendix 6. Model fairness assessment with regards to sex and race/ethnicity variables

This supplemental material has been provided by the authors to give readers additional information about their work.

© 2025 Srivastava T et al. *JAMA Health Forum*.

eAppendix 1. Patient identification and ICD-10-CM inclusion codes by stimulant-involved overdose outcome.			
**Outcome**	**ICD-10-CM code in any position**	**Plus a 6th character of**	**Plus a 7th character of**
**Cocaine overdose**	T40.5X: Poisoning by cocaine	1: Accidental (unintentional)<br>2: Intentional self-harm<br>3: Assault<br>4: Undetermined intent<br><br>Excluding:<br>5: Adverse effect<br>6: Underdosing	A: Initial encounter<br><br>Excluding:<br>D: Subsequent encounter<br>S: Sequela
**Overdose due to other stimulants**	T43.60: Poisoning by unspecified psychostimulants<br>T43.62: Poisoning by amphetamines, accidental (unintentional), initial encounter<br>T43.64: Poisoning by ecstasy<br>T43.69: Poisoning by other psychostimulants	1: Accidental (unintentional)<br>2: Intentional self-harm<br>3: Assault<br>4: Undetermined intent<br><br>Excluding:<br>5: Adverse effect<br>6: Underdosing	A: Initial encounter<br><br>Excluding:<br>D: Subsequent encounter<br>S: Sequela
**Opioid overdose (for the determination of stimulant overdose type ONLY, NOT for stimulant overdose identification)**	T40.0X: Poisoning by opium<br>T40.1X: Poisoning by heroin<br>T40.2X: Poisoning by other opioids<br>T40.3X: Poisoning by methadone<br>T40.4X: Poisoning by synthetic narcotics<br>T40.60: Poisoning by unspecified narcotics<br>T40.69: Poisoning by other narcotics	1: Accidental (unintentional)<br>2: Intentional self-harm<br>3: Assault<br>4: Undetermined intent<br><br>Excluding:<br>5: Adverse effect<br>6: Underdosing	A: Initial encounter<br><br>Excluding:<br>D: Subsequent encounter<br>S: Sequela

© 2025 Srivastava T et al. *JAMA Health Forum*.

eAppendix 2. Detailed information about the study methods.

Individual-level candidate variables from Medicaid files. The following variables are individual-level variables extracted from the Medicaid data set. They were included in the initial LASSO variable selection model and a subset were used in the final parsimonious prediction model. A full list with detailed descriptions of the variables is at the end of this Appendix 2.

Category	Variables
Demographics	age, sex, race/ethnicity, HHS region, dual eligibility, comprehensive managed care plan, behavioral health managed care plan, basis of Medicaid eligibility, citizenship status, any disability, household size, immigration status, income relative to federal poverty level, marital status, primary language spoken, temporary assistance for needy families
Behavioral and mental health disorders in the baseline year	anxiety, depression, schizophrenia, manic episode, bipolar disorder, PTSD, ADHD, personality and behavioral disorders, sleep disorders
Substance-related disorders in the baseline year	alcohol-related disorders, cannabis-related disorders, cocaine-related disorders, hallucinogen-related disorders, inhalant-related disorders, nicotine-related disorders, opioid use disorder, other psychoactive substance-related disorders, sedative/hypnotic/anxiolytic-related disorders, stimulant-related disorders

Area-level variables.

American Community Survey (ACS) variables included those related to income, education, employment, housing, household characteristics, transportation, and social demographics (age, race, sex). For the American Community Survey (ACS) variables, the sampling year of Medicaid data corresponds to the last year of the 5-year data collection period. We also included the following area-level socioeconomic inequality indicators: Gini Index, rural-urban classification, 2015 Social Deprivation Index scores, retail opioid dispensing rates, and health resources (such as the access to and availability of health care, facilities, and providers). For the Social Deprivation Index (SDI), the 2015 SDI score was linked to all sampling years (2015-2020). For opioid dispensing rate, the rate for one year before the sampling year was linked to each sampling year (e.g., opioid dispensing rate in 2015 was linked to the sampling year of 2016). For the Area Health Resources Files (AHRF) variables, the most recent available data before the sampling year was linked to each sampling year (e.g., doctor density was available for 2015 and 2018, thus the 2015 data were linked to sampling years of 2016-2018, and the 2018 data were linked to sampling years of 2019-2020). A full list with detailed descriptions of the variables is at the end of this Appendix 2.

Cohort and model development.

We allowed patients to contribute case events to more than one sampling year. However, we did include only the ICD-10 diagnosis codes for overdose having a 7th character of “A” (initial encounter) and did not include 7th characters of “D” (subsequent encounter) or “S” (sequela).

© 2025 Srivastava T et al. *JAMA Health Forum*.

Also, we collapsed ED and inpatient admissions that were separated by fewer than two days into a single event. Recognizing that the COVID-19 pandemic was a disruptive force in healthcare, 2020 was selected as the *test* year rather than included as a *development* year. 2020 was also the latest data year at the time of analyses, and we intentionally chose this as the *test* year hypothesizing that the model should be overly pessimistic given those disruptions. We included “missing” as a separate category where it was applicable in the individual-level data, in order to use the whole data set. For example, for the race variable we had an indicator for Missing vs. non-Hispanic White (reference). For the area-level variables, we excluded patients with missing zip code (~1%), as well as patients missing any of the continuous covariates (~3.7%).

**Full List of Individual and Area-level Candidate Predictors**
Gini index
Percent population less than 100% FPL: population under 0.99/total population
Percentage of total households receiving food stamps/SNAP in the past 12 months
Percent population 25 years or more with less than 12 years of education: population with less than high school diploma or 12 years of education/total population
Percentage of adults ages 25-44 with some post-secondary education
Percent non-employed (not in labor force + unemployed) / (civilian + not in the labor force) for the population 16-64 years
Percent population living in renter-occupied housing units (Renter occupied housing units/ (Owner-occupied housing units + Renter occupied housing units))
Percent population living in crowded housing units (Tenure by Occupants Per Room Â– a population with = 1.01 occupants per room in Owner-occupied housing units and Renter occupied housing units) / total population
Percent population living in high-density housing (structures with 10 or more units)
The proportion of homeowner households that are estimated to spend at least 35% of their income on their mortgage
The proportion of renter households that are estimated to spend at least 35% of their income on their rent
Percentage of vacant homes
Percent single-parent households with dependents < 18 years: (total single-parent households (male and female) with dependents <18 years)/total population)
Percent households with female heads and children < 18 years old
Percent population with no car (population with no vehicle available/total population)
Percentage of the workforce that usually drives alone to work: number of workers who commute alone to work via car, truck, or van / total workforce
Percentage of workers who drive alone with a commute longer than 30 minutes: workers who drive alone (via car, truck, or van) for more than 30 minutes during their commute / number of workers who drive alone (via car, truck, or van) during their commute.
(population under 5 years of age + women between the ages of 15-44 years + everyone 65 years and over)/total population
Percent under 25 years of age
Percent ages 25-64
Percent over 65 years of age
Percent male

© 2025 Srivastava T et al. *JAMA Health Forum*.

Full List of Individual and Area-level Candidate Predictors
Percent non-Hispanic White
Percent living with a disability
Percent of total population that is foreign born
Percent of total population remaining in the same residence for the past 5 years
Percentage of population under age 65 without health insurance: number of people with no health insurance coverage under the age of 65/ number of people under age 65
Employment rate of workforce employed in manufacturing jobs per 1,000 residents
Employment rate of workforce employed in agriculture jobs per 1,000 residents
Employment rate of workforce employed in mining job per 1,000 residents
Employment rate of workforce employed in professional/service jobs per 1,000 residents
Median household income in the past 12 months
Median value (dollars)
Total population per square mile land area
Social deprivation index (range 1-100)
Retail opioid prescriptions dispensed per 100 persons per year; available years: 2015-2020
Total medical doctors per 100,000 population; available years: 2015 and 2018
Total specialists per 100,000 population; available years: 2015 and 2018
Total primary care physicians per 100,000 population; available years: 2015, 2016, 2017, and 2018
Total hospitals per 100,000 population; available years: 2015 and 2018
Total federally qualified helth centers per 100,000 population; available years: 2015, 2016, 2017, 2018, and 2019
Total hospital beds per 100,000 population; available years: 2015 and 2018
Sampling year
patient ID number
Age (years) on January 1 of sampling year
Case (1=yes, 0=no)
member of the subcohort (1=yes, 0=no)
Antibiotics (during the calendar year preceding the sampling year)
Antidepressants (during the calendar year preceding the sampling year)
Antipsychotics (during the calendar year preceding the sampling year)
Antiretroviral agents (during the calendar year preceding the sampling year)
Anxiolytics (during the calendar year preceding the sampling year)
Buprenorphine (during the calendar year preceding the sampling year)
Gabapentinoids (during the calendar year preceding the sampling year)
Treatments for hepatitis C (during the calendar year preceding the sampling year)
Methadone (during the calendar year preceding the sampling year)
Mood stabilizing agents (during the calendar year preceding the sampling year)
Muscle relaxants (during the calendar year preceding the sampling year)
Naltrexone (during the calendar year preceding the sampling year)
Opioids (during the calendar year preceding the sampling year)
Sedative-hypnotics (during the calendar year preceding the sampling year)
Stimulants (during the calendar year preceding the sampling year)

© 2025 Srivastava T et al. *JAMA Health Forum*.

Full List of Individual and Area-level Candidate Predictors
number of ED visits in baseline year
was patient in Comprehensive Managed Care during baseline year
was patient in Behavioral Health Comprehensive Managed Care during baseline year
patient dually eligible for Medicaid and Medicare
patient number
Gender: female (reference is Male)
Race: Black (reference is non-Hispanic White)
Race: Other (reference is non-Hispanic White)
Race: Hispanic (reference is non-Hispanic White)
Race: Missing (reference is non-Hispanic White)
US Region: Midwest (reference is West)
US Region: Northeast (reference is West)
US Region: Southeast (reference is West)
US Region: Southwest (reference is West)
Any disability
Household size: 2 people (reference is 1 person)
Household size: 3 people (reference is 1 person)
Household size: 4+ people (reference is 1 person)
Household size: missing (reference is 1 person)
Immigration status: qualified non-citizen (reference is U.S. citizen)
Immigration status: missing (reference is U.S. citizen)
Income relative to federal poverty line: => 101 (reference is 0 to 100% of the FPL)
Income relative to federal poverty line: missing (reference is 0 to 100% of the FPL)
Marital status: married (reference is Never married/partnered)
Marital status: divorced (reference is Never married/partnered)
Marital status: other (reference is Never married/partnered)
Marital status: missing (reference is Never married/partnered)
Primary language spoken: other (reference is English)
Primary language spoken: missing (reference is English)
Temporary Assistance for Needy Families: did receive benefits (reference is Individual did not receive TANF benefits)
Temporary Assistance for Needy Families: missing (reference is Individual did not receive TANF benefits)
Basis of Medicaid eligibility: Child (reference is Income)
Basis of Medicaid eligibility: Disability (reference is Income)
Basis of Medicaid eligibility: Other (reference is Income)
Basis of Medicaid eligibility: missing (reference is Income)
Citizenship status: non-citizen (reference is U.S. citizen)
Citizenship status: missing (reference is U.S. citizen)
Degree of urbanization and adjacency to a metro area (2010 version): small rural (reference is Urban)
Degree of urbanization and adjacency to a metro area (2010 version): large rural (reference is Urban)
Degree of urbanization and adjacency to a metro area (2010 version): isolated rural (reference is Urban)
Indicator for primary care shortage: None (reference is part of county designated as shortage area)

© 2025 Srivastava T et al. *JAMA Health Forum*.

Full List of Individual and Area-level Candidate Predictors
Indicator for primary care shortage: Whole (reference is part of county designated as shortage area)
Indicator for mental health shortage: None (reference is part of county designated as shortage area)
Indicator for mental health shortage: Whole (reference is part of county designated as shortage area)
Hallucinogen-related disorders: any position IP, OT, or LT diagnosis during baseline year (reference is No evidence)
Inhalant-related disorders: any position IP, OT, or LT diagnosis during baseline year (reference is No evidence)
Hepatitus B: any position IP, OT, or LT diagnosis during baseline year (reference is No evidence)
HIV: any position IP, OT, or LT diagnosis during baseline year (reference is No evidence)
Infectious endocarditis: any position IP, OT, or LT diagnosis during baseline year (reference is No evidence)
Anxiety: any position IP diagnosis during baseline year (reference is No evidence)
Anxiety: any position OT or LT diagnosis during baseline year (reference is No evidence)
Manic episode: any position IP diagnosis during baseline year (reference is No evidence)
Manic episode: any position OT or LT diagnosis during baseline year
PTSD: any position IP diagnosis during baseline year (reference is No evidence)
PTSD: any position OT or LT diagnosis during baseline year (reference is No evidence)
ADHD: any position IP diagnosis during baseline year (reference is No evidence)
ADHD: any position OT or LT diagnosis during baseline year (reference is No evidence)
Personality disorders: any position IP diagnosis during baseline year (reference is No evidence)
Personality disorders: any position OT or LT diagnosis during baseline year (reference is No evidence)
Sleep disorders: any position IP diagnosis during baseline year (reference is No evidence)
Sleep disorders: any position OT or LT diagnosis during baseline year (reference is No evidence)
Cannabis: any position IP diagnosis during baseline year (reference is No evidence)
Cannabis: any position OT or LT diagnosis during baseline year (reference is No evidence)
Cocaine: any position IP diagnosis during baseline year (reference is No evidence)
Cocaine: any position OT or LT diagnosis during baseline year (reference is No evidence)
Nicotine: any position IP diagnosis during baseline year (reference is No evidence)
Nicotine: any position OT or LT diagnosis during baseline year (reference is No evidence)
Other psychoactive substance: any position IP diagnosis during baseline year (reference is No evidence)
Other psychoactive substance: any position OT or LT diagnosis during baseline year (reference is No evidence)
Sedative/hypnotics/anxiolytic: any position IP diagnosis during baseline year (reference is No evidence)
Sedative/hypnotics/anxiolytic: any position OT or LT diagnosis during baseline year (reference is No evidence)
Stimulants: any position IP diagnosis during baseline year (reference is No evidence)
Stimulants: any position OT or LT diagnosis during baseline year (reference is No evidence)
Depression disorder: primary IP diagnosis during baseline year (reference is No evidence)
Depression: non-primary IP diagnosis during baseline year (reference is No evidence)
Depression disorder: any OT or LT diagnosis during baseline year (reference is No evidence)
Schizophrenia: primary IP diagnosis during baseline year (reference is No evidence)
Schizophrenia: non-primary IP diagnosis during baseline year (reference is No evidence)
Schizophrenia: any OT or LT diagnosis during baseline year (reference is No evidence)
Bipolar disorder: primary IP diagnosis during baseline year (reference is No evidence)
Bipolar disorder: non-primary IP diagnosis during baseline year (reference is No evidence)

© 2025 Srivastava T et al. *JAMA Health Forum*.

Full List of Individual and Area-level Candidate Predictors
Bipolar disorder: any OT or LT diagnosis during baseline year (reference is No evidence)
Alcohol disorder: primary IP diagnosis during baseline year (reference is No evidence)
Alcohol disorder: non-primary IP diagnosis during baseline year (reference is No evidence)
Alcohol disorder: any OT or LT diagnosis during baseline year (reference is No evidence)
Opioid use disorder disorder: primary IP diagnosis during baseline year (reference is No evidence)
Opioid use disorder disorder: non-primary IP diagnosis during baseline year (reference is No evidence)
Opioid use disorder disorder: any OT or LT diagnosis during baseline year (reference is No evidence)
Cardiovascular: any position IP diagnosis during baseline year (reference is No evidence)
Cardiovascular: any position OT or LT diagnosis during baseline year (reference is No evidence)
Asthma: any position IP diagnosis during baseline year (reference is No evidence)
Asthma: any position OT or LT diagnosis during baseline year (reference is No evidence)
Lung disease: any position IP diagnosis during baseline year (reference is No evidence)
Lung disease: any position OT or LT diagnosis during baseline year (reference is No evidence)
HTN: any position IP diagnosis during baseline year (reference is No evidence)
HTN: any position OT or LT diagnosis during baseline year (reference is No evidence)
CKD: any position IP diagnosis during baseline year (reference is No evidence)
CKD: any position OT or LT diagnosis during baseline year (reference is No evidence)
Hepatitis C: any position IP diagnosis during baseline year (reference is No evidence)
Hepatitis C: any position OT or LT diagnosis during baseline year (reference is No evidence)
Abscess or cellulitis: any position IP diagnosis during baseline year (reference is No evidence)
Abscess or cellulitis: any position OT or LT diagnosis during baseline year (reference is No evidence)
Sexually transmitted infections: any position IP diagnosis during baseline year (reference is No evidence)
Sexually transmitted infections: any position OT or LT diagnosis during baseline year (reference is No evidence)

© 2025 Srivastava T et al. *JAMA Health Forum*.

eAppendix 3. TRIPOD Checklist: Prediction Model Development

Section/Topic	Item	Checklist Item	Page
**Title and abstract**			
Title	1	Identify the study as developing and/or validating a multivariable prediction model, the target population, and the outcome to be predicted.	1
Abstract	2	Provide a summary of objectives, study design, setting, participants, sample size, predictors, outcome, statistical analysis, results, and conclusions.	3
**Introduction**			
Background and objectives	3a	Explain the medical context (including whether diagnostic or prognostic) and rationale for developing or validating the multivariable prediction model, including references to existing models.	4-5
	3b	Specify the objectives, including whether the study describes the development or validation of the model or both.	5
**Methods**			
Source of data	4a	Describe the study design or source of data (e.g., randomized trial, cohort, or registry data), separately for the development and validation data sets, if applicable.	5-8
	4b	Specify the key study dates, including start of accrual; end of accrual; and, if applicable, end of follow-up.	6
Participants	5a	Specify key elements of the study setting (e.g., primary care, secondary care, general population) including number and location of centres.	5-6
	5b	Describe eligibility criteria for participants.	6
	5c	Give details of treatments received, if relevant.	-
Outcome	6a	Clearly define the outcome that is predicted by the prediction model, including how and when assessed.	6-7
	6b	Report any actions to blind assessment of the outcome to be predicted.	-
Predictors	7a	Clearly define all predictors used in developing or validating the multivariable prediction model, including how and when they were measured.	7
	7b	Report any actions to blind assessment of predictors for the outcome and other predictors.	-
Sample size	8	Explain how the study size was arrived at.	5-6
Missing data	9	Describe how missing data were handled (e.g., complete-case analysis, single imputation, multiple imputation) with details of any imputation method.	6
Statistical analysis methods	10a	Describe how predictors were handled in the analyses.	8
	10b	Specify type of model, all model-building procedures (including any predictor selection), and method for internal validation.	7-9
	10d	Specify all measures used to assess model performance and, if relevant, to compare multiple models.	9-11, 13
Risk groups	11	Provide details on how risk groups were created, if done.	9
**Results**			
Participants	13a	Describe the flow of participants through the study, including the number of participants with and without the outcome and, if applicable, a summary of the follow-up time. A diagram may be helpful.	9
	13b	Describe the characteristics of the participants (basic demographics, clinical features, available predictors), including the number of participants with missing data for predictors and outcome.	22, Appen dix 4
Model development	14a	Specify the number of participants and outcome events in each analysis.	9, 22, Appen dix 4
	14b	If done, report the unadjusted association between each candidate predictor and outcome.	-
Model specification	15a	Present the full prediction model to allow predictions for individuals (i.e., all regression coefficients, and model intercept or baseline survival at a given time point).	9-11, 24-27, Appen dix 5
	15b	Explain how to the use the prediction model.	-
Model performance	16	Report performance measures (with CIs) for the prediction model.	9-11
**Discussion**			

© 2025 Srivastava T et al. *JAMA Health Forum*.

Limitations	18	Discuss any limitations of the study (such as nonrepresentative sample, few events per predictor, missing data).	14-15
Interpretation	19b	Give an overall interpretation of the results, considering objectives, limitations, and results from similar studies, and other relevant evidence.	12-15
Implications	20	Discuss the potential clinical use of the model and implications for future research.	15
**Other information**			
Supplementary information	21	Provide information about the availability of supplementary resources, such as study protocol, Web calculator, and data sets.	16, Supple ment
Funding	22	Give the source of funding and the role of the funders for the present study.	16

© 2025 Srivastava T et al. *JAMA Health Forum*.

eAppendix 4. Additional individual-level sociodemographic characteristics of Medicaid claims data (2016-2020) used for development and testing by outcome. A) Cocaine-involved with opioid involvement, B) Cocaine-involved without opioid involvement, C) Methamphetamines, ecstasy, or other psychostimulant-involved with opioid involvement.

Characteristics	Development (2016-2019)		Testing (2020)	
	Cases	Subcohort	Cases	Subcohort
**A. Cocaine-involved with opioid involvement**				
	(n=6,547)	(n=216,552)	(n=1,912)	(n=62,004)
**Sampling year**				
2016	815 (12.4)	26,465 (12.2)	2020 (100%)	
2017	1,952 (29.8)	62,929 (29.1)		
2018	1,944 (29.7)	64,563 (29.8)		
2019	1,836 (28.0)	62,595 (28.9)		
**Age (years): mean (SD)**	41.5 (12.5)	41.6 (20.2)	42.6 (12.8)	42.1 (20.4)
**Sex**				
Female	2,892 (44.2)	128,236 (59.2)	760 (39.7)	36,465 (58.8)
Male	3,655 (55.8)	88,316 (40.8)	1,152 (60.3)	25,539 (41.2)
**Race**				
White, non-Hispanic	2,945 (45.0)	83,766 (38.7)	854 (44.7)	24,844 (40.1)
Black, non-Hispanic	1,524 (23.3)	39,838 (18.4)	586 (30.6)	11,731 (18.9)
Other	104 (1.6)	15,706 (7.3)	33 (1.7)	4,946 (8.0)
Hispanic, all races	527 (8.0)	39,572 (18.3)	179 (9.4)	12,109 (19.5)
Missing	1,447 (22.1)	37,670 (17.4)	260 (13.6)	8,374 (13.5)
**Household size**				
1 person	2,570 (39.3)	46,782 (21.6)	759 (39.7)	13,374 (21.6)
2 people	191 (2.9)	8,842 (4.1)	67 (3.5)	2,613 (4.2)
3 people	96 (1.5)	7,067 (3.3)	33 (1.7)	2,160 (3.5)
4 or more people	154 (2.4)	13,766 (6.4)	33 (1.7)	4,261 (6.9)
Missing	3,536 (54.0)	140,095 (64.7)	1,020 (53.3)	39,596 (63.9)
**Income relative to federal poverty level (FPL)**				
0 to 100% of the FPL	3,452 (52.7)	70,163 (32.4)	984 (51.5)	20,961 (33.8)
≥ 101% of the FPL	275 (4.2)	15,701 (7.3)	83 (4.3)	4,480 (7.2)
Missing	2,820 (43.1)	130,688 (60.3)	845 (44.2)	36,563 (59.0)
**Citizenship status**				
Non-citizen	80 (1.2)	14,864 (6.9)	10 (0.5)	4,446 (7.2)
U.S. Citizen	4,572 (69.8)	154,076 (71.1)	1,525 (79.8)	48,015 (77.4)
Missing	1,895 (28.9)	47,612 (22.0)	377 (19.7)	9,543 (15.4)
**HHS region**				
Midwest	1,684 (25.7)	49,404 (22.8)	361 (18.9)	12,505 (20.2)
Northeast	3,232 (49.4)	42,257 (19.5)	1,102 (57.6)	14,320 (23.1)
Southeast	966 (14.8)	41,609 (19.2)	210 (11.0)	10,209 (16.5)
Southwest	192 (2.9)	14,654 (6.8)	48 (2.5)	4,117 (6.6)
West	473 (7.2)	68,628 (31.7)	191 (10.0)	20,853 (33.6)
**Dually eligible (Yes)**	799 (12.2)	50,655 (23.4)	188 (9.8)	14,399 (23.2)

© 2025 Srivastava T et al. *JAMA Health Forum*.

**Comprehensive managed care plan (Yes)**	5,096 (77.8)	161,791 (74.7)	1,509 (78.9)	47,317 (76.3)
**Behavioral health managed care plan (Yes)**	1,017 (15.5)	24,690 (11.4)	300 (15.7)	5,194 (8.4)
**Basis of Medicaid eligibility**				
Child	171 (2.6)	33,856 (15.6)	40 (2.1)	9,699 (15.6)
Disability	1,080 (16.5)	52,811 (24.4)	275 (14.4)	13,759 (22.2)
Income	5,111 (78.1)	117,165 (54.1)	1,543 (80.7)	34,941 (56.4)
Other	116 (1.8)	9,869 (4.6)	41 (2.1)	2,943 (4.7)
Missing	69 (1.1)	2,851 (1.3)	13 (0.7)	662 (1.1)
**Any disability (Yes)**	1,695 (25.9)	39,150 (18.1)	426 (22.3)	10,557 (17.0)

B. Cocaine-involved without opioid involvement

Characteristics	Development (2016-2019)		Testing (2020)	
	Cases	Subcohort	Cases	Subcohort
	(n=22,460)	(n=216,552)	(n=6,746)	(n=62,004)
**Sampling year**				
2016	2,414 (10.7)	26,465 (12.2)	2020 (100%)	
2017	6,280 (28.0)	62,929 (29.1)		
2018	6,647 (29.6)	64,563 (29.8)		
2019	7,119 (31.7)	62,595 (28.9)		
**Age (years): mean (SD)**	47.5 (12.7)	41.6 (20.2)	48.6 (13.1)	42.1 (20.4)
**Sex**				
Female	9,404 (41.9)	128,236 (59.2)	2,771 (41.1)	36,465 (58.8)
Male	13,056 (58.1)	88,316 (40.8)	3,975 (58.9)	25,539 (41.2)
**Race**				
White, non-Hispanic	5,351 (23.8)	83,766 (38.7)	1,651 (24.5)	24,844 (40.1)
Black, non-Hispanic	10,452 (46.5)	39,838 (18.4)	3,391 (50.3)	11,731 (18.9)
Other	393 (1.7)	15,706 (7.3)	124 (1.8)	4,946 (8.0)
Hispanic, all races	1,932 (8.6)	39,572 (18.3)	683 (10.1)	12,109 (19.5)
Missing	4,332 (19.3)	37,670 (17.4)	897 (13.3)	8,374 (13.5)
**Household size**				
1 person	6,847 (30.5)	46,782 (21.6)	2,161 (32.0)	13,374 (21.6)
2 people	445 (2.0)	8,842 (4.1)	143 (2.1)	2,613 (4.2)
3 people	269 (1.2)	7,067 (3.3)	96 (1.4)	2,160 (3.5)
4 or more people	483 (2.2)	13,766 (6.4)	165 (2.4)	4,261 (6.9)
Missing	14,416 (64.2)	140,095 (64.7)	4,181 (62.0)	39,596 (63.9)
**Income relative to federal poverty level (FPL)**				
0 to 100% of the FPL	15,782 (70.3)	158,383 (73.1)	2,861 (42.4)	20,961 (33.8)
≥ 101% of the FPL	261 (1.2)	10,801 (5.0)	177 (2.6)	4,480 (7.2)
Missing	6,417 (28.6)	47,368 (21.9)	3,708 (55.0)	36,563 (59.0)
**Citizenship status**				
Non-citizen	337 (1.5)	14,864 (6.9)	79 (1.2)	4,446 (7.2)
U.S. Citizen	15,635 (69.6)	154,076 (71.1)	5,206 (77.2)	48,015 (77.4)
Missing	6,488 (28.9)	47,612 (22.0)	1,461 (21.7)	9,543 (15.4)
**HHS region**				

© 2025 Srivastava T et al. *JAMA Health Forum*.

Midwest	5,235 (23.3)	49,404 (22.8)	1,314 (19.5)	12,505 (20.2)
Northeast	7,292 (32.5)	42,257 (19.5)	2,594 (38.5)	14,320 (23.1)
Southeast	5,000 (22.3)	41,609 (19.2)	1,226 (18.2)	10,209 (16.5)
Southwest	1,585 (7.1)	14,654 (6.8)	442 (6.6)	4,117 (6.6)
West	3,348 (14.9)	68,628 (31.7)	1,170 (17.3)	20,853 (33.6)
**Dually eligible (Yes)**	3,678 (16.4)	50,655 (23.4)	1,002 (14.9)	14,399 (23.2)
**Comprehensive managed care plan (Yes)**	16,692 (74.3)	161,791 (74.7)	5,087 (75.4)	47,317 (76.3)
**Behavioral health managed care plan (Yes)**	3,057 (13.6)	24,690 (11.4)	860 (12.7)	5,194 (8.4)
**Basis of Medicaid eligibility**				
Child	528 (2.4)	33,856 (15.6)	159 (2.4)	9,699 (15.6)
Disability	3,318 (14.8)	52,811 (24.4)	847 (12.6)	13,759 (22.2)
Income	17,871 (79.6)	117,165 (54.1)	5,554 (82.3)	34,941 (56.4)
Other	477 (2.1)	9,869 (4.6)	141 (2.1)	2,943 (4.7)
Missing	266 (1.2)	2,851 (1.3)	45 (0.7)	662 (1.1)
**Any disability (Yes)**	8,247 (36.7)	39,150 (18.1)	2,290 (33.9)	10,557 (17.0)

**C. Methamphetamines, ecstasy, or other psychostimulant-involved with opioid involvement**				
**Characteristics**	**Development (2016-2019)**		**Testing (2020)**	
	**Cases**	**Subcohort**	**Cases**	**Subcohort**
	(n=4,908)	(n=216,552)	(n=1,724)	(n=62,004)
**Sampling year**				
2016	638 (13.0)	26,465 (12.2)	2020 (100%)	
2017	1,409 (28.7)	62,929 (29.1)		
2018	1,392 (28.4)	64,563 (29.8)		
2019	1,469 (29.9)	62,595 (28.9)		
**Age (years): mean (SD)**	37.3 (11.7)	41.6 (20.2)	37.6 (11.6)	42.1 (20.4)
**Sex**				
Female	2,269 (46.2)	128,236 (59.2)	726 (42.1)	36,465 (58.8)
Male	2,639 (53.8)	88,316 (40.8)	998 (57.9)	25,539 (41.2)
**Race**				
White, non-Hispanic	3,136 (63.9)	83,766 (38.7)	1,144 (66.4)	24,844 (40.1)
Black, non-Hispanic	299 (6.1)	39,838 (18.4)	126 (7.3)	11,731 (18.9)
Other	227 (4.6)	15,706 (7.3)	89 (5.2)	4,946 (8.0)
Hispanic, all races	455 (9.3)	39,572 (18.3)	168 (9.7)	12,109 (19.5)
Missing	791 (16.1)	37,670 (17.4)	197 (11.4)	8,374 (13.5)
**Household size**				
1 person	1,879 (38.3)	46,782 (21.6)	624 (36.2)	13,374 (21.6)
2 people	169 (3.4)	8,842 (4.1)	50 (2.9)	2,613 (4.2)
3 people	91 (1.9)	7,067 (3.3)	36 (2.1)	2,160 (3.5)
4 or more people	154 (3.1)	13,766 (6.4)	35 (2.0)	4,261 (6.9)
Missing	2,615 (53.3)	140,095 (64.7)	979 (56.8)	39,596 (63.9)
**Income relative to federal poverty level (FPL)**				
0 to 100% of the FPL	1,850 (37.7)	70,163 (32.4)	669 (38.8)	20,961 (33.8)
≥ 101% of the FPL	101 (2.1)	15,701 (7.3)	38 (2.2)	4,480 (7.2)

© 2025 Srivastava T et al. *JAMA Health Forum*.

Missing	2,957 (60.2)	130,688 (60.3)	1,017 (59.0)	36,563 (59.0)
**Citizenship status**				
Non-citizen	67 (1.4)	14,864 (6.9)	19 (1.1)	4,446 (7.2)
U.S. Citizen	4,030 (82.1)	154,076 (71.1)	1,480 (85.8)	48,015 (77.4)
Missing	811 (16.5)	47,612 (22.0)	225 (13.1)	9,543 (15.4)
**HHS region**				
Midwest	900 (18.3)	49,404 (22.8)	305 (17.7)	12,505 (20.2)
Northeast	405 (8.3)	42,257 (19.5)	232 (13.5)	14,320 (23.1)
Southeast	1,134 (23.1)	41,609 (19.2)	343 (19.9)	10,209 (16.5)
Southwest	293 (6.0)	14,654 (6.8)	82 (4.8)	4,117 (6.6)
West	2,176 (44.3)	68,628 (31.7)	762 (44.2)	20,853 (33.6)
**Dually eligible (Yes)**	433 (8.8)	50,655 (23.4)	119 (6.9)	14,399 (23.2)
**Comprehensive managed care plan (Yes)**	3,976 (81.0)	161,791 (74.7)	1,417 (82.2)	47,317 (76.3)
**Behavioral health managed care plan (Yes)**	989 (20.2)	24,690 (11.4)	150 (8.7)	5,194 (8.4)
**Basis of Medicaid eligibility**				
Child	171 (3.5)	33,856 (15.6)	62 (3.6)	9,699 (15.6)
Disability	832 (17.0)	52,811 (24.4)	232 (13.5)	13,759 (22.2)
Income	3,735 (76.1)	117,165 (54.1)	1,388 (80.5)	34,941 (56.4)
Other	101 (2.1)	9,869 (4.6)	32 (1.9)	2,943 (4.7)
Missing	69 (1.4)	2,851 (1.3)	10 (0.6)	662 (1.1)
**Any disability (Yes)**	1,018 (20.7)	39,150 (18.1)	285 (16.5)	10,557 (17.0)

© 2025 Srivastava T et al. *JAMA Health Forum*.

eAppendix 5. Additional Weighted Cox model hazard ratio results with LASSO-selected predictors by outcome. A) Cocaine-involved with opioid involvement, B) Cocaine-involved without opioid involvement, C) Methamphetamines, ecstasy, or other psychostimulant-involved with opioid involvement.

A. Cocaine-involved with opioid involvement

Variable	LASSO Coefficient	Hazard Ratio (HR)	HR 95% Confidence Interval (CI)		Chi-squared P-value
Individual-level characteristics					
Age (years)	-0.01	0.99	0.99	1.00	<0.001
Gender: Female (ref: Male)	-0.41	0.66	0.63	0.70	<0.001
Race: Other (ref: NH White)	-0.57	0.57	0.46	0.70	<0.001
Race: Hispanic (ref: NH White)	-0.08	0.92	0.83	1.02	0.127
Race: Missing (ref: NH White)	0.12	1.13	1.06	1.20	<0.001
U.S. Region: Midwest (ref: West)	0.66	1.94	1.67	2.26	<0.001
U.S. Region: Northeast (ref: West)	1.36	3.90	3.38	4.51	<0.001
U.S. Region: Southeast (ref: West)	0.88	2.41	2.08	2.80	<0.001
U.S. Region: Southwest (ref: West)	0.58	1.78	1.46	2.17	<0.001
Household size: 2 people (Ref: 1 person)	-0.41	0.66	0.57	0.77	<0.001
Household size: 3 people (Ref: 1 person)	-0.67	0.51	0.41	0.63	<0.001
Household size: 4+ people (Ref: 1 person)	-0.59	0.55	0.47	0.66	<0.001
Immigration status: Missing (Ref: U.S. citizen)	0.66	1.93	1.73	2.15	<0.001
Income relative to federal poverty line (FPL): ≥101% (Ref: 0-100% of the FPL)	-0.42	0.66	0.57	0.75	<0.001
Income relative to federal poverty line (FPL): Missing (Ref: 0-100% of the FPL)	-0.24	0.79	0.74	0.85	<0.001
Marital status: Married (Ref: Never married/partnered)	-0.64	0.53	0.44	0.63	<0.001
Marital status: Divorced (Ref: Never married/partnered)	-0.09	0.91	0.82	1.01	0.080
Marital status: Other (Ref: Never married/partnered)	-0.22	0.80	0.66	0.98	0.030
Marital status: Missing (Ref: Never married/partnered)	-0.21	0.81	0.75	0.87	<0.001
Primary language spoken: Other (Ref: English)	-0.65	0.52	0.43	0.64	<0.001
Primary language spoken: Missing (Ref: English)	-0.13	0.87	0.81	0.94	<0.001
Temporary Assistance for Needy Families (TANF): Did receive benefits (Ref: Individual did not receive TANF benefits)	0.14	1.15	1.03	1.28	0.010
Temporary Assistance for Needy Families (TANF): Missing (Ref: Individual did not receive TANF benefits)	0.34	1.40	1.31	1.51	<0.001
Patient dually eligible for Medicaid and Medicare	-0.45	0.64	0.58	0.70	<0.001
Basis of Medicaid eligibility: Child (Ref: Income)	-1.27	0.28	0.24	0.33	<0.001
Basis of Medicaid eligibility: Disability (Ref: Income)	-0.25	0.78	0.73	0.84	<0.001
Basis of Medicaid eligibility: Other (Ref: Income)	-0.40	0.67	0.56	0.81	<0.001

© 2025 Srivastava T et al. *JAMA Health Forum*.

Citizenship status: Non-citizen (Ref: U.S. citizen)	-0.58	0.56	0.45	0.71	<0.001
Citizenship status: Missing (Ref: U.S. citizen)	-0.52	0.59	0.53	0.66	<0.001
Area-level characteristics					
Gini index	1.25	4.51	2.08	9.78	<0.001
Percentage of total households receiving food stamps/SNAP in the past 12 months	0.83	1.74	0.93	3.25	0.082
Percentage of adults ages 25-44 with some post-secondary education	-2.87	2.19	1.51	3.18	<0.001
Percent population living in renter-occupied housing units	1.66	1.02	0.69	1.51	0.937
Percent population living in crowded housing units	2.88	1.23	0.33	4.55	0.755
Percent population living in high-density housing (structures with 10 or more units)	0.51	1.15	0.86	1.54	0.334
Proportion of renter households that are estimated to spend at least 35% of their income on their rent	-0.95	2.43	1.67	3.53	<0.001
Percentage of vacant homes	0.41	1.36	0.88	2.09	0.163
Percent households with female heads and children <18 years old	-0.16	1.37	0.65	2.86	0.404
Percent population with no car	-0.93	0.73	0.44	1.21	0.219
Percentage of workers who drive alone with a commute longer than 30 minutes	1.36	1.07	0.84	1.38	0.583
Percent of population with high need (under 5 years of age + women between the ages of 15-44 years + everyone 65 years and over)	0.69	0.38	0.10	1.53	0.174
Percent under 25 years of age	-2.10	0.12	0.05	0.33	<0.001
Percent ages 25-64	-1.08	0.34	0.11	1.05	0.061
Percent male	1.68	5.37	1.42	20.35	0.013
Percent Hispanic White (ref: Non-Hispanic White)	-0.49	0.61	0.50	0.75	<0.001
Percent living with a disability	0.65	1.93	0.66	5.66	0.234
Percent of total population that is foreign born	-0.44	0.64	0.42	0.98	0.040
Percentage of population under age 65 without health insurance	-0.32	0.73	0.30	1.75	0.478
Employment rate of workforce employed in manufacturing jobs per 1,000 residents	0.00	1.00	1.00	1.00	0.005
Employment rate of workforce employed in agriculture jobs per 1,000 residents	0.00	1.00	1.00	1.00	0.004
Employment rate of workforce employed in professional/service jobs per 1,000 residents	0.00	1.00	1.00	1.00	0.010
Median value (dollars)	0.00	1.00	1.00	1.00	<0.001
Total population per square mile land area	0.00	1.00	1.00	1.00	0.041
Total specialists per 100,000 population; available years: 2015 and 2018	0.00	1.00	1.00	1.00	<0.001
Total primary care physicians per 100,000 population	0.00	1.00	1.00	1.00	0.002
Total hospitals per 100,000 population	-0.04	0.96	0.93	0.99	0.007
Total federally qualified health centers per 100,000 population	-0.03	0.97	0.96	0.99	<0.001
Degree of urbanization and adjacency to a metro area (2010 version): small rural (Ref: Urban)	-0.08	0.92	0.75	1.14	0.458
Degree of urbanization and adjacency to a metro area (2010 version): large rural (Ref: Urban)	-0.15	0.86	0.76	0.97	0.017

© 2025 Srivastava T et al. *JAMA Health Forum*.

Degree of urbanization and adjacency to a metro area (2010 version): isolated rural (Ref: Urban)	-0.43	0.65	0.48	0.88	0.005
Indicator for primary care shortage: Whole (Ref: Part of county designated as shortage area)	-0.37	0.69	0.57	0.85	<0.001
Indicator for mental care shortage: Whole (Ref: Part of county designated as shortage area)	-0.27	0.76	0.70	0.84	<0.001
Individual-level clinical characteristics					
Antidepressants	0.05	1.05	0.98	1.12	0.152
Antipsychotics	-0.08	0.92	0.86	0.99	0.034
Antiretrovirals	-0.39	0.68	0.54	0.84	<0.001
Anxiolytics	0.34	1.41	1.32	1.50	<0.001
Buprenorphine	-0.12	0.89	0.82	0.96	0.004
Gabapentinoids	0.19	1.21	1.13	1.29	<0.001
Treatments for hepatitis C	0.16	1.17	0.96	1.43	0.115
Methadone	0.24	1.28	1.17	1.39	<0.001
Mood stabilizing agents	-0.20	0.82	0.74	0.91	<0.001
Naltrexone	0.36	1.44	1.27	1.63	<0.001
Opioids	0.18	1.20	1.13	1.27	<0.001
Sedative-hypnotics	-0.11	0.90	0.84	0.96	0.002
Stimulants	-0.14	0.87	0.76	0.98	0.027
Number of ED visits in baseline year	0.01	1.01	1.00	1.01	<0.001
Was patient in Comprehensive Managed Care during baseline year	0.10	1.10	1.03	1.19	0.007
Was patient in Behavioral Health Comprehensive Managed Care during baseline year	0.11	1.12	1.03	1.22	0.008
Any disability	0.15	1.16	1.09	1.24	<0.001
Individual-level clinical diagnoses					
Abscess or cellulitis: Any position IP diagnosis during baseline year (Ref: No evidence)	0.41	1.51	1.34	1.70	<0.001
Abscess or cellulitis: Any position OT or LT diagnosis during baseline year (Ref: No evidence)	0.39	1.48	1.37	1.61	<0.001
ADHD: Any position IP diagnosis during baseline year (Ref: No evidence)	0.09	1.09	0.90	1.33	0.380
ADHD: Any position OT or LT diagnosis during baseline year (Ref: No evidence)	-0.09	0.91	0.79	1.06	0.229
Alcohol disorder: Any position OT or LT diagnosis during baseline year (Ref: No evidence)	0.55	1.73	1.61	1.87	<0.001
Alcohol disorder: Non-primary IP diagnosis during baseline year (Ref: No evidence)	0.27	1.31	1.18	1.44	<0.001
Alcohol disorder: Primary IP diagnosis during baseline year (Ref: No evidence)	-0.20	0.82	0.71	0.95	0.009
Anxiety: Any position IP diagnosis during baseline year (Ref: No evidence)	0.09	1.10	0.99	1.22	0.076
Anxiety: Any position OT or LT diagnosis during baseline year (Ref: No evidence)	0.04	1.04	0.97	1.12	0.264
Asthma: Any position IP diagnosis during baseline year (Ref: No evidence)	0.30	1.35	1.22	1.50	<0.001
Asthma: Any position OT or LT diagnosis during baseline year (Ref: No evidence)	0.14	1.15	1.04	1.26	0.006

© 2025 Srivastava T et al. *JAMA Health Forum*.

Bipolar disorder: Any position OT or LT diagnosis during baseline year (Ref: No evidence)	0.23	1.25	1.15	1.37	<0.001
Bipolar disorder: Non-primary IP diagnosis during baseline year (Ref: No evidence)	0.35	1.42	1.25	1.60	<0.001
Bipolar disorder: Primary IP diagnosis during baseline year (Ref: No evidence)	0.15	1.16	0.98	1.36	0.085
Cannabis: Any position IP diagnosis during baseline year (Ref: No evidence)	-0.20	0.82	0.72	0.92	0.001
Cannabis: Any position OT or LT diagnosis during baseline year (Ref: No evidence)	0.05	1.05	0.95	1.18	0.346
Cardiovascular: Any position IP diagnosis during baseline year (Ref: No evidence)	-0.29	0.75	0.66	0.86	<0.001
Cardiovascular: Any position OT or LT diagnosis during baseline year (Ref: No evidence)	-0.09	0.92	0.82	1.03	0.135
Chronic kidney disease: Any position IP diagnosis during baseline year (Ref: No evidence)	0.23	1.26	1.13	1.40	<0.001
Chronic kidney disease: Any position OT or LT diagnosis during baseline year (Ref: No evidence)	-0.15	0.86	0.77	0.97	0.013
Cocaine: Any position IP diagnosis during baseline year (Ref: No evidence)	1.56	4.74	4.30	5.23	<0.001
Cocaine: Any position OT or LT diagnosis during baseline year (Ref: No evidence)	1.27	3.57	3.27	3.90	<0.001
Depression disorder: Any position OT or LT diagnosis during baseline year (Ref: No evidence)	0.19	1.21	1.13	1.30	<0.001
Depression disorder: Non-primary IP diagnosis during baseline year (Ref: No evidence)	0.14	1.15	1.03	1.28	0.010
Depression disorder: Primary IP diagnosis during baseline year (Ref: No evidence)	0.25	1.29	1.13	1.47	<0.001
Hallucinogen-related disorders: Any position IP, OT, or LT diagnosis during baseline year (Ref: No evidence)	0.46	1.58	1.23	2.03	<0.001
Hepatitis C: Any position IP diagnosis during baseline year (Ref: No evidence)	0.09	1.09	0.96	1.25	0.194
Hepatitis C: Any position OT or LT diagnosis during baseline year (Ref: No evidence)	0.37	1.45	1.31	1.60	<0.001
HIV: Any position IP, OT, or LT diagnosis during baseline year (Ref: No evidence)	0.44	1.55	1.27	1.89	<0.001
Hypertension: Any position IP diagnosis during baseline year (Ref: No evidence)	-0.15	0.86	0.78	0.95	0.003
Hypertension: Any position OT or LT diagnosis during baseline year (Ref: No evidence)	-0.12	0.88	0.82	0.95	0.001
Infectious endocarditis: Any position IP, OT, or LT diagnosis during baseline year (Ref: No evidence)	-0.12	0.88	0.71	1.10	0.266
Inhalant-related disorders: Any position IP, OT, or LT diagnosis during baseline year (Ref: No evidence)	0.55	1.73	1.39	2.16	<0.001
Lung disease: Any position IP diagnosis during baseline year (Ref: No evidence)	0.21	1.23	1.09	1.39	<0.001
Lung disease: Any position OT or LT diagnosis during baseline year (Ref: No evidence)	0.37	1.44	1.32	1.58	<0.001
Manic episode: Any position IP diagnosis during baseline year (Ref: No evidence)	-0.54	0.58	0.45	0.76	<0.001
Manic episode: Any position OT or LT diagnosis during baseline year (Ref: No evidence)	0.18	1.20	1.01	1.42	0.039
Nicotine: Any position IP diagnosis during baseline year (Ref: No evidence)	0.21	1.23	1.13	1.35	<0.001
Nicotine: Any position OT or LT diagnosis during baseline year (Ref: No evidence)	0.31	1.37	1.27	1.48	<0.001
Opioid use disorder: Any position OT or LT diagnosis during baseline year (Ref: No evidence)	2.08	7.98	7.40	8.60	<0.001
Opioid use disorder: Non-primary IP diagnosis during baseline year (Ref: No evidence)	2.39	10.94	9.87	12.13	<0.001
Opioid use disorder: Primary IP diagnosis during baseline year (Ref: No evidence)	2.59	13.35	11.70	15.23	<0.001
Other psychoactive substance: Any position IP diagnosis during baseline year (Ref: No evidence)	0.95	2.58	2.29	2.89	<0.001
Other psychoactive substance: Any position OT or LT diagnosis during baseline year (Ref: No evidence)	0.48	1.61	1.50	1.74	<0.001

© 2025 Srivastava T et al. *JAMA Health Forum*.

Personality disorders Any position IP diagnosis during baseline year (Ref: No evidence)	0.18	1.20	1.01	1.42	0.039
PTSD: Any position IP diagnosis during baseline year (Ref: No evidence)	0.00	1.00	0.88	1.14	0.998
PTSD: Any position OT or LT diagnosis during baseline year (Ref: No evidence)	-0.09	0.92	0.83	1.02	0.114
Schizophrenia: Any position OT or LT diagnosis during baseline year (Ref: No evidence)	-0.05	0.95	0.87	1.04	0.260
Schizophrenia: Primary IP diagnosis during baseline year (Ref: No evidence)	0.05	1.05	0.89	1.25	0.562
Sedative/hypnotics/anxiolytic: Any position IP diagnosis during baseline year (Ref: No evidence)	0.06	1.06	0.92	1.22	0.432
Sedative/hypnotics/anxiolytic: Any position OT or LT diagnosis during baseline year (Ref: No evidence)	0.27	1.31	1.13	1.53	<0.001
Sexually transmitted infections: Any position IP diagnosis during baseline year (Ref: No evidence)	-0.12	0.89	0.65	1.22	0.458
Sexually transmitted infections: Any position OT or LT diagnosis during baseline year (Ref: No evidence)	0.32	1.37	1.20	1.57	<0.001
Sleep disorders: Any position IP diagnosis during baseline year (Ref: No evidence)	-0.10	0.91	0.79	1.04	0.171
Sleep disorders: Any position OT or LT diagnosis during baseline year (Ref: No evidence)	-0.24	0.79	0.71	0.88	<0.001
Stimulants: Any position IP diagnosis during baseline year (Ref: No evidence)	-0.82	0.44	0.37	0.53	<0.001
Stimulants: Any position OT or LT diagnosis during baseline year (Ref: No evidence)	-0.15	0.86	0.73	1.01	0.069
**B. Cocaine-involved without opioid involvement**					
**Variable**	**LASSO Coefficient**	**Hazard Ratio (HR)**	**HR 95% Confidence Interval (CI)**		**Chi-squared P-value**
Individual-level characteristics					
Age (years)	0.00	1.00	1.00	1.00	<0.001
Gender: Female (ref: Male)	-0.47	0.63	0.61	0.65	<0.001
Race: Black (ref: NH White)	0.65	1.91	1.83	1.99	<0.001
Race: Other (ref: NH White)	-0.40	0.67	0.60	0.74	<0.001
Race: Hispanic (ref: NH White)	-0.03	0.97	0.91	1.03	0.290
Race: Missing (ref: NH White)	0.37	1.45	1.38	1.51	<0.001
U.S. Region: Midwest (ref: West)	0.12	1.13	1.04	1.22	0.003
U.S. Region: Northeast (ref: West)	0.42	1.52	1.40	1.64	<0.001
U.S. Region: Southeast (ref: West)	0.54	1.72	1.60	1.85	<0.001
U.S. Region: Southwest (ref: West)	0.19	1.21	1.11	1.32	<0.001
Household size: 2 people (Ref: 1 person)	-0.37	0.69	0.62	0.76	<0.001
Household size: 3 people (Ref: 1 person)	-0.40	0.67	0.59	0.76	<0.001
Household size: 4+ people (Ref: 1 person)	-0.49	0.61	0.55	0.68	<0.001
Household size: Missing (Ref: 1 person)	0.05	1.05	1.01	1.10	0.026
Immigration status: Non-citizen (Ref: U.S. citizen)	1.11	3.05	2.33	3.99	<0.001
Immigration status: Missing (Ref: U.S. citizen)	0.72	2.05	1.93	2.19	<0.001
Income relative to federal poverty line (FPL): ≥101% (Ref: 0-100% of the FPL)	-0.52	0.60	0.55	0.65	<0.001

© 2025 Srivastava T et al. *JAMA Health Forum*.

Income relative to federal poverty line (FPL): Missing (Ref: 0-100% of the FPL)	-0.16	0.86	0.82	0.89	<0.001
Marital status: Married (Ref: Never married/partnered)	-0.42	0.66	0.59	0.73	<0.001
Marital status: Divorced (Ref: Never married/partnered)	-0.14	0.87	0.81	0.92	<0.001
Marital status: Other (Ref: Never married/partnered)	-0.04	0.97	0.89	1.05	0.422
Marital status: Missing (Ref: Never married/partnered)	-0.12	0.88	0.85	0.92	<0.001
Primary language spoken: Other (Ref: English)	-0.78	0.46	0.41	0.51	<0.001
Primary language spoken: Missing (Ref: English)	-0.36	0.70	0.67	0.73	<0.001
Temporary Assistance for Needy Families (TANF): Did receive benefits (Ref: Individual did not receive TANF benefits)	0.07	1.07	1.01	1.13	0.019
Temporary Assistance for Needy Families (TANF): Missing (Ref: Individual did not receive TANF benefits)	0.20	1.22	1.17	1.28	<0.001
Patient dually eligible for Medicaid and Medicare	-0.62	0.54	0.51	0.56	<0.001
Basis of Medicaid eligibility: Child (Ref: Income)	-1.13	0.32	0.29	0.35	<0.001
Basis of Medicaid eligibility: Disability (Ref: Income)	-0.30	0.74	0.71	0.78	<0.001
Basis of Medicaid eligibility: Other (Ref: Income)	-0.19	0.83	0.75	0.91	<0.001
Basis of Medicaid eligibility: Missing (Ref: Income)	0.29	1.34	1.17	1.53	<0.001
Citizenship status: Non-citizen (Ref: U.S. citizen)	-1.87	0.16	0.12	0.20	<0.001
Citizenship status: Missing (Ref: U.S. citizen)	-0.35	0.70	0.66	0.75	<0.001
Area-level characteristics					
Gini index	3.00	20.10	12.73	31.73	<0.001
Percent population less than 100% FPL	-0.32	0.73	0.47	1.15	0.171
Percentage of total households receiving food stamps/SNAP in the past 12 months	-1.63	0.20	0.14	0.27	<0.001
Percent population 25 years or more with less than 12 years of education	2.03	7.60	5.05	11.46	<0.001
Percentage of adults ages 25-44 with some post-secondary education	0.66	1.94	1.52	2.48	<0.001
Percent non-employed	-1.69	0.19	0.13	0.27	<0.001
Percent population living in renter-occupied housing units	-1.05	0.35	0.27	0.46	<0.001
Percent population living in crowded housing units	0.78	2.18	1.18	4.04	0.013
Percent population living in high-density housing (structures with 10 or more units)	0.64	1.91	1.64	2.22	<0.001
Proportion of homeowner households that are estimated to spend at least 35% of their income on their mortgage	0.59	1.80	1.44	2.26	<0.001
Proportion of renter households that are estimated to spend at least 35% of their income on their rent	1.56	4.74	3.80	5.91	<0.001
Percentage of vacant homes	0.26	1.30	1.00	1.69	0.054
Percent households with female heads and children <18 years old	0.56	1.75	1.19	2.58	0.004
Percent population with no car	1.11	3.03	2.13	4.32	<0.001
Percentage of the workforce that usually drives alone to work	1.01	2.74	2.10	3.56	<0.001

© 2025 Srivastava T et al. *JAMA Health Forum*.

Percentage of workers who drive alone with a commute longer than 30 minutes	0.68	1.98	1.72	2.28	<0.001
Percent of population with high need (under 5 years of age + women between the ages of 15-44 years + everyone 65 years and over)	3.73	41.77	18.04	96.73	<0.001
Percent under 25 years of age	0.85	2.33	1.30	4.19	0.005
Percent ages 25-64	1.27	3.56	1.71	7.40	0.001
Percent male	4.62	101.28	46.65	219.91	<0.001
Percent non-Hispanic (NH) White	-0.68	0.51	0.45	0.57	<0.001
Percent of total population that is foreign born	-1.66	0.19	0.15	0.24	<0.001
Percent of total population remaining in the same residence for the past 5 years	0.32	1.38	1.01	1.89	0.046
Percentage of population under age 65 without health insurance	1.43	4.19	2.70	6.52	<0.001
Employment rate of workforce employed in manufacturing jobs per 1,000 residents	0.00	1.00	1.00	1.00	0.005
Employment rate of workforce employed in agriculture jobs per 1,000 residents	0.00	1.00	1.00	1.00	0.018
Employment rate of workforce employed in mining job per 1,000 residents	0.00	1.00	1.00	1.00	0.028
Employment rate of workforce employed in professional/service jobs per 1,000 residents	0.00	1.00	1.00	1.00	0.006
Median household income in the past 12 months	0.00	1.00	1.00	1.00	0.131
Median value (dollars)	0.00	1.00	1.00	1.00	<0.001
Total population per square mile land area	0.00	1.00	1.00	1.00	<0.001
Social deprivation index	0.01	1.01	1.00	1.01	<0.001
Retail opioid prescriptions dispensed per 100 persons per year	0.00	1.00	1.00	1.00	0.447
Total medical doctors per 100,000 population	0.00	1.00	1.00	1.00	<0.001
Total specialists per 100,000 population	0.00	1.00	1.00	1.00	<0.001
Total primary care physicians per 100,000 population	0.00	1.00	1.00	1.00	<0.001
Total hospitals per 100,000 population	-0.06	0.94	0.93	0.96	<0.001
Total federally qualified health centers per 100,000 population	-0.02	0.98	0.97	0.99	<0.001
Total hospital beds per 100,000 population	0.00	1.00	1.00	1.00	<0.001
Degree of urbanization and adjacency to a metro area (2010 version): small rural (ref: Urban)	-0.10	0.91	0.80	1.03	0.125
Degree of urbanization and adjacency to a metro area (2010 version): large rural (ref: Urban)	-0.08	0.92	0.85	0.99	0.028
Degree of urbanization and adjacency to a metro area (2010 version): isolated rural (ref: Urban)	-0.54	0.58	0.49	0.70	<0.001
Indicator for primary care shortage: None (Ref: Part of county designated as shortage area)	0.17	1.19	1.08	1.30	0.000
Indicator for mental health shortage: None (Ref: Part of county designated as shortage area)	-0.14	0.87	0.80	0.95	0.002
Indicator for mental health shortage: Whole (Ref: Part of county designated as shortage area)	-0.22	0.81	0.76	0.85	<0.001
Individual-level clinical characteristics					
Antibiotics	0.02	1.02	0.99	1.06	0.231
Antidepressants	0.05	1.05	1.01	1.09	0.012

© 2025 Srivastava T et al. *JAMA Health Forum*.

Antiretrovirals	-0.72	0.49	0.44	0.54	<0.001
Buprenorphine	-0.15	0.86	0.80	0.93	<0.001
Gabapentinoids	0.16	1.17	1.12	1.21	<0.001
Treatments for hepatitis C	0.19	1.21	1.04	1.41	0.011
Methadone	0.32	1.37	1.28	1.48	<0.001
Mood stabilizing agents	-0.31	0.73	0.69	0.77	<0.001
Naltrexone	0.32	1.37	1.25	1.52	<0.001
Opioids	0.35	1.42	1.38	1.47	<0.001
Sedative-hypnotics	0.08	1.08	1.04	1.12	<0.001
Stimulants	-0.14	0.87	0.80	0.94	0.000
Number of ED visits in baseline year	0.01	1.01	1.01	1.01	<0.001
Was patient in Comprehensive Managed Care during baseline year	0.00	1.00	0.96	1.04	0.849
Was patient in Behavioral Health Comprehensive Managed Care during baseline year	0.23	1.26	1.20	1.33	<0.001
Any disability	0.23	1.26	1.22	1.30	<0.001
Individual-level clinical diagnoses					
Abscess or cellulitis: Any position IP diagnosis during baseline year (Ref: No evidence)	-0.14	0.87	0.81	0.94	0.000
Abscess or cellulitis: Any position OT or LT diagnosis during baseline year (Ref: No evidence)	0.14	1.15	1.09	1.21	<0.001
ADHD: Any position IP diagnosis during baseline year (Ref: No evidence)	-0.93	0.40	0.34	0.47	<0.001
Alcohol disorder: Any position OT or LT diagnosis during baseline year (Ref: No evidence)	0.60	1.82	1.74	1.90	<0.001
Alcohol disorder: Non-primary IP diagnosis during baseline year (Ref: No evidence)	0.39	1.48	1.41	1.56	<0.001
Alcohol disorder: Primary IP diagnosis during baseline year (Ref: No evidence)	0.18	1.19	1.10	1.29	<0.001
Anxiety: Any position IP diagnosis during baseline year (Ref: No evidence)	0.09	1.09	1.04	1.15	0.001
Anxiety: Any position OT or LT diagnosis during baseline year (Ref: No evidence)	0.05	1.05	1.01	1.10	0.026
Asthma: Any position IP diagnosis during baseline year (Ref: No evidence)	0.38	1.46	1.39	1.53	<0.001
Asthma: Any position OT or LT diagnosis during baseline year (Ref: No evidence)	0.20	1.22	1.16	1.28	<0.001
Bipolar disorder: Any position OT or LT diagnosis during baseline year (Ref: No evidence)	0.32	1.38	1.31	1.45	<0.001
Bipolar disorder: Non-primary IP diagnosis during baseline year (Ref: No evidence)	0.41	1.50	1.41	1.60	<0.001
Bipolar disorder: Primary IP diagnosis during baseline year (Ref: No evidence)	0.29	1.34	1.22	1.46	<0.001
Cannabis: Any position IP diagnosis during baseline year (Ref: No evidence)	-0.17	0.85	0.80	0.90	<0.001
Cannabis: Any position OT or LT diagnosis during baseline year (Ref: No evidence)	0.07	1.07	1.01	1.15	0.033
Cardiovascular: Any position IP diagnosis during baseline year (Ref: No evidence)	0.82	2.27	2.17	2.38	<0.001
Cardiovascular: Any position OT or LT diagnosis during baseline year (Ref: No evidence)	0.48	1.62	1.54	1.71	<0.001
Chronic kidney disease: Any position IP diagnosis during baseline year (Ref: No evidence)	0.56	1.75	1.68	1.83	<0.001
Chronic kidney disease: Any position OT or LT diagnosis during baseline year (Ref: No evidence)	0.13	1.13	1.08	1.19	<0.001

© 2025 Srivastava T et al. *JAMA Health Forum*.

Cocaine: Any position IP diagnosis during baseline year (Ref: No evidence)	2.44	11.51	10.95	12.09	<0.001
Cocaine: Any position OT or LT diagnosis during baseline year (Ref: No evidence)	2.02	7.57	7.20	7.95	<0.001
Depression disorder: Any position OT or LT diagnosis during baseline year (Ref: No evidence)	0.15	1.16	1.12	1.21	<0.001
Depression disorder: Non-primary IP diagnosis during baseline year (Ref: No evidence)	0.04	1.04	0.98	1.10	0.172
Depression disorder: Primary IP diagnosis during baseline year (Ref: No evidence)	0.30	1.36	1.26	1.46	<0.001
Hallucinogen-related disorders: Any position IP, OT, or LT diagnosis during baseline year (Ref: No evidence)	0.77	2.16	1.90	2.44	<0.001
Hepatitis B: Any position IP, OT, or LT diagnosis during baseline year (Ref: No evidence)	0.05	1.05	0.90	1.22	0.531
Hepatitis C: Any position IP diagnosis during baseline year (Ref: No evidence)	-0.21	0.81	0.75	0.88	<0.001
Hepatitis C: Any position OT or LT diagnosis during baseline year (Ref: No evidence)	0.06	1.06	0.99	1.14	0.084
HIV: Any position IP, OT, or LT diagnosis during baseline year (Ref: No evidence)	0.90	2.45	2.23	2.69	<0.001
Hypertension: Any position IP diagnosis during baseline year (Ref: No evidence)	0.03	1.03	0.99	1.08	0.164
Hypertension: Any position OT or LT diagnosis during baseline year (Ref: No evidence)	0.16	1.17	1.13	1.22	<0.001
Infectious endocarditis: Any position IP, OT, or LT diagnosis during baseline year (Ref: No evidence)	-0.40	0.67	0.58	0.78	<0.001
Inhalant-related disorders: Any position IP, OT, or LT diagnosis during baseline year (Ref: No evidence)	0.31	1.37	1.19	1.57	<0.001
Lung disease: Any position IP diagnosis during baseline year (Ref: No evidence)	0.46	1.58	1.51	1.66	<0.001
Lung disease: Any position OT or LT diagnosis during baseline year (Ref: No evidence)	0.40	1.49	1.42	1.56	<0.001
Manic episode: Any position IP diagnosis during baseline year (Ref: No evidence)	0.26	1.30	1.13	1.49	0.000
Manic episode: Any position OT or LT diagnosis during baseline year (Ref: No evidence)	0.60	1.82	1.67	1.98	<0.001
Nicotine: Any position IP diagnosis during baseline year (Ref: No evidence)	0.54	1.71	1.63	1.79	<0.001
Nicotine: Any position OT or LT diagnosis during baseline year (Ref: No evidence)	0.51	1.66	1.59	1.74	<0.001
Opioid use disorder: Any position OT or LT diagnosis during baseline year (Ref: No evidence)	0.50	1.65	1.57	1.73	<0.001
Opioid use disorder: Non-primary IP diagnosis during baseline year (Ref: No evidence)	0.20	1.22	1.15	1.31	<0.001
Opioid use disorder: Primary IP diagnosis during baseline year (Ref: No evidence)	0.37	1.45	1.32	1.61	<0.001
Other psychoactive substance: Any position IP diagnosis during baseline year (Ref: No evidence)	0.81	2.24	2.11	2.39	<0.001
Other psychoactive substance: Any position OT or LT diagnosis during baseline year (Ref: No evidence)	0.30	1.35	1.28	1.41	<0.001
Personality disorders: Any position IP diagnosis during baseline year (Ref: No evidence)	-0.04	0.96	0.87	1.06	0.382
Personality disorders: Any position OT or LT diagnosis during baseline year (Ref: No evidence)	-0.24	0.78	0.72	0.86	<0.001
PTSD: Any position IP diagnosis during baseline year (Ref: No evidence)	0.34	1.41	1.30	1.52	<0.001
PTSD: Any position OT or LT diagnosis during baseline year (Ref: No evidence)	0.15	1.16	1.09	1.23	<0.001
Schizophrenia: Any position OT or LT diagnosis during baseline year (Ref: No evidence)	0.12	1.12	1.07	1.18	<0.001
Schizophrenia: Non-primary IP diagnosis during baseline year (Ref: No evidence)	-0.01	0.99	0.92	1.06	0.746
Schizophrenia: Primary IP diagnosis during baseline year (Ref: No evidence)	0.33	1.39	1.29	1.49	<0.001
Sedative/hypnotics/anxiolytic: Any position IP diagnosis during baseline year (Ref: No evidence)	0.05	1.05	0.95	1.17	0.313

© 2025 Srivastava T et al. *JAMA Health Forum*.

Sedative/hypnotics/anxiolytic: Any position OT or LT diagnosis during baseline year (Ref: No evidence)	-0.25	0.78	0.68	0.89	0.000
Sexually transmitted infections: Any position IP diagnosis during baseline year (Ref: No evidence)	-0.31	0.73	0.63	0.85	<0.001
Sexually transmitted infections: Any position OT or LT diagnosis during baseline year (Ref: No evidence)	0.27	1.31	1.21	1.41	<0.001
Sleep disorders: Any position IP diagnosis during baseline year (Ref: No evidence)	-0.25	0.78	0.73	0.83	<0.001
Sleep disorders: Any position OT or LT diagnosis during baseline year (Ref: No evidence)	-0.17	0.84	0.80	0.89	<0.001
Stimulants: Any position IP diagnosis during baseline year (Ref: No evidence)	-0.22	0.80	0.73	0.89	<0.001
Stimulants: Any position OT or LT diagnosis during baseline year (Ref: No evidence)	0.18	1.20	1.10	1.32	<0.001

**C. Methamphetamines, ecstasy, or other psychostimulant-involved with opioid involvement**					
**Variable**	**LASSO Coefficient**	**Hazard Ratio (HR)**	**HR 95% Confidence Interval (CI)**		**Chi-squared P-value**
Individual-level characteristics					
Age (years)	-0.02	0.98	0.98	0.98	<0.001
Gender: Female (ref: Male)	-0.40	0.67	0.63	0.71	<0.001
Race: Black (ref: NH White)	-0.80	0.45	0.40	0.51	<0.001
Race: Other (ref: NH White)	-0.22	0.80	0.70	0.93	0.003
Race: Hispanic (ref: NH White)	-0.42	0.66	0.59	0.73	<0.001
Race: Missing (ref: NH White)	-0.19	0.83	0.76	0.90	<0.001
Household size: 2 people (Ref: 1 person)	-0.41	0.66	0.56	0.78	<0.001
Household size: 3 people (Ref: 1 person)	-0.43	0.65	0.52	0.81	<0.001
Household size: 4+ people (Ref: 1 person)	-0.37	0.69	0.58	0.82	<0.001
Household size: Missing (Ref: 1 person)	-0.13	0.88	0.81	0.95	0.002
Immigration status: Qualified non-citizen (Ref: U.S. citizen)	-0.39	0.67	0.41	1.10	0.117
Immigration status: Missing (Ref: U.S. citizen)	0.39	1.48	1.27	1.73	<0.001
Income relative to federal poverty line (FPL): ≥101% (Ref: 0-100% of the FPL)	-0.67	0.51	0.42	0.63	<0.001
Income relative to federal poverty line (FPL): Missing (Ref: 0-100% of the FPL)	0.00	1.00	0.93	1.08	0.978
Marital status: Married (Ref: Never married/partnered)	-0.42	0.66	0.53	0.82	<0.001
Marital status: Divorced (Ref: Never married/partnered)	0.08	1.08	0.97	1.20	0.176
Marital status: Missing (Ref: Never married/partnered)	-0.12	0.89	0.82	0.96	0.003
Primary language spoken: Other (Ref: English)	-1.84	0.16	0.11	0.24	<0.001
Primary language spoken: Missing (Ref: English)	-0.15	0.86	0.78	0.95	0.004
Temporary Assistance for Needy Families (TANF): Did receive benefits (Ref: Individual did not receive TANF benefits)	-0.29	0.75	0.66	0.85	<0.001
Temporary Assistance for Needy Families (TANF): Missing (Ref: Individual did not receive TANF benefits)	0.48	1.62	1.47	1.78	<0.001
Patient dually eligible for Medicaid and Medicare	-0.31	0.73	0.65	0.83	<0.001

© 2025 Srivastava T et al. *JAMA Health Forum*.

Basis of Medicaid eligibility: Child (Ref: Income)	-1.42	0.24	0.21	0.29	<0.001
Basis of Medicaid eligibility: Disability (Ref: Income)	-0.32	0.73	0.67	0.79	<0.001
Basis of Medicaid eligibility: Other (Ref: Income)	-0.28	0.76	0.62	0.93	0.008
Basis of Medicaid eligibility: Missing (Ref: Income)	-0.20	0.82	0.63	1.06	0.134
Citizenship status: Non-citizen (Ref: U.S. citizen)	-0.66	0.52	0.36	0.75	<0.001
Citizenship status: Missing (Ref: U.S. citizen)	-0.44	0.64	0.55	0.75	<0.001
Area-level characteristics					
Percentage of total households receiving food stamps/SNAP in the past 12 months	-0.05	0.95	0.51	1.76	0.868
Percentage of adults ages 25-44 with some post-secondary education	-0.46	0.63	0.43	0.92	0.018
Percent non-employed (not in labor force + unemployed) / (civilian + not in the labor force) for the population 16-64 years	-1.59	0.20	0.11	0.37	<0.001
Percent population living in crowded housing units	2.22	9.22	3.17	26.82	<0.001
Percent population living in high-density housing (structures with 10 or more units)	0.59	1.81	1.35	2.43	<0.001
Proportion of homeowner households that are estimated to spend at least 35% of their income on their mortgage	-0.66	0.52	0.32	0.83	0.006
Proportion of renter households that are estimated to spend at least 35% of their income on their rent	0.37	1.45	1.00	2.10	0.051
Percentage of vacant homes	-0.10	0.90	0.58	1.42	0.659
Percentage of workers who drive alone with a commute longer than 30 minutes	-0.47	0.62	0.49	0.80	<0.001
Percent under 25 years of age	-0.36	0.70	0.33	1.47	0.344
Percent male	3.80	44.68	11.80	169.19	<0.001
Percent Hispanic White (ref: Non-Hispanic White)	0.52	1.68	1.35	2.09	<0.001
Percent living with a disability	3.22	25.01	9.59	65.19	<0.001
Percent of total population remaining in the same residence for the past 5 years	0.10	1.11	0.68	1.81	0.680
Percentage of population under age 65 without health insurance	-0.83	0.43	0.19	0.98	0.045
Employment rate of workforce employed in agriculture jobs per 1,000 residents	0.00	1.00	1.00	1.00	0.001
Employment rate of workforce employed in mining job per 1,000 residents	0.00	1.00	1.00	1.00	0.384
Employment rate of workforce employed in professional/service jobs per 1,000 residents	0.00	1.00	1.00	1.00	0.016
Total specialists per 100,000 population	0.00	1.00	1.00	1.00	0.633
Total federally qualified health centers per 100,000 population	0.00	1.00	0.99	1.00	0.180
U.S. Region: Midwest (ref: West)	-0.97	0.38	0.34	0.43	<0.001
U.S. Region: Northeast (ref: West)	-1.53	0.22	0.19	0.25	<0.001
U.S. Region: Southeast (ref: West)	-0.11	0.90	0.81	0.99	0.035
Indicator for primary care shortage: Whole (Ref: Part of county designated as shortage area)	-0.10	0.91	0.79	1.05	0.186
Indicator for mental care shortage: None (Ref: Part of county designated as shortage area)	0.01	1.01	0.88	1.17	0.866
Indicator for mental care shortage: Whole (Ref: Part of county designated as shortage area)	0.12	1.12	1.04	1.22	0.004

© 2025 Srivastava T et al. *JAMA Health Forum*.

Individual-level clinical characteristics					
Antibiotics	-0.16	0.85	0.80	0.92	<0.001
Antipsychotics	0.16	1.18	1.08	1.28	<0.001
Anxiolytics	0.30	1.36	1.26	1.46	<0.001
Gabapentinoids	0.27	1.32	1.22	1.42	<0.001
Treatments for hepatitis C	0.09	1.09	0.82	1.46	0.564
Methadone	0.53	1.70	1.53	1.90	<0.001
Mood stabilizing agents	-0.25	0.78	0.69	0.88	<0.001
Muscle relaxants	0.23	1.26	1.17	1.37	<0.001
Naltrexone	-0.08	0.92	0.78	1.09	0.328
Opioids	0.18	1.19	1.11	1.28	<0.001
Stimulants	0.28	1.33	1.18	1.49	<0.001
Number of ED visits in baseline year	0.01	1.01	1.01	1.02	<0.001
Was patient in Comprehensive Managed Care during baseline year	0.11	1.12	1.03	1.23	0.012
Was patient in Behavioral Health Comprehensive Managed Care during baseline year	0.03	1.03	0.94	1.13	0.552
Any disability	-0.21	0.81	0.75	0.88	<0.001
Individual-level clinical diagnoses					
Abscess or cellulitis: Any position IP diagnosis during baseline year (Ref: No evidence)	0.27	1.31	1.13	1.51	<0.001
Abscess or cellulitis: Any position OT or LT diagnosis during baseline year (Ref: No evidence)	0.48	1.62	1.48	1.76	<0.001
ADHD: Any position IP diagnosis during baseline year (Ref: No evidence)	0.70	2.01	1.61	2.52	<0.001
Alcohol disorder: Any position OT or LT diagnosis during baseline year (Ref: No evidence)	0.51	1.67	1.52	1.83	<0.001
Alcohol disorder: Non-primary IP diagnosis during baseline year (Ref: No evidence)	0.35	1.41	1.24	1.62	<0.001
Alcohol disorder: Primary IP diagnosis during baseline year (Ref: No evidence)	-0.46	0.63	0.48	0.83	<0.001
Anxiety: Any position OT or LT diagnosis during baseline year (Ref: No evidence)	0.15	1.16	1.08	1.25	<0.001
Asthma: Any position IP diagnosis during baseline year (Ref: No evidence)	-0.19	0.83	0.71	0.98	0.026
Asthma: Any position OT or LT diagnosis during baseline year (Ref: No evidence)	-0.26	0.77	0.68	0.88	<0.001
Bipolar disorder: Any position OT or LT diagnosis during baseline year (Ref: No evidence)	0.22	1.25	1.12	1.40	<0.001
Bipolar disorder: Non-primary IP diagnosis during baseline year (Ref: No evidence)	0.11	1.12	0.94	1.33	0.222
Bipolar disorder: Primary IP diagnosis during baseline year (Ref: No evidence)	0.03	1.03	0.82	1.28	0.806
Cannabis: Any position IP diagnosis during baseline year (Ref: No evidence)	-0.40	0.67	0.56	0.81	<0.001
Cannabis: Any position OT or LT diagnosis during baseline year (Ref: No evidence)	-0.08	0.92	0.80	1.06	0.254
Cardiovascular: Any position IP diagnosis during baseline year (Ref: No evidence)	-0.30	0.74	0.62	0.89	0.001
Cardiovascular: Any position OT or LT diagnosis during baseline year (Ref: No evidence)	-0.16	0.85	0.73	1.01	0.057

© 2025 Srivastava T et al. *JAMA Health Forum*.

Chronic kidney disease: Any position IP diagnosis during baseline year (Ref: No evidence)	0.45	1.57	1.37	1.79	<0.001
Chronic kidney disease: Any position OT or LT diagnosis during baseline year (Ref: No evidence)	-0.12	0.89	0.76	1.03	0.109
Cocaine: Any position IP diagnosis during baseline year (Ref: No evidence)	-0.11	0.89	0.74	1.08	0.236
Cocaine: Any position OT or LT diagnosis during baseline year (Ref: No evidence)	-0.11	0.90	0.76	1.06	0.212
Depression disorder: Non-primary IP diagnosis during baseline year (Ref: No evidence)	0.33	1.39	1.22	1.57	<0.001
Depression disorder: Primary IP diagnosis during baseline year (Ref: No evidence)	0.38	1.46	1.24	1.72	<0.001
Hallucinogen-related disorders: Any position IP, OT, or LT diagnosis during baseline year (Ref: No evidence)	0.90	2.47	1.65	3.71	<0.001
Hepatitis B: Any position IP, OT, or LT diagnosis during baseline year (Ref: No evidence)	-0.11	0.89	0.61	1.31	0.559
Hepatitis C: Any position IP diagnosis during baseline year (Ref: No evidence)	0.14	1.16	0.97	1.37	0.098
Hepatitis C: Any position OT or LT diagnosis during baseline year (Ref: No evidence)	0.48	1.62	1.43	1.83	<0.001
Hypertension: Any position IP diagnosis during baseline year (Ref: No evidence)	-0.13	0.88	0.77	1.00	0.048
Hypertension: Any position OT or LT diagnosis during baseline year (Ref: No evidence)	-0.19	0.83	0.76	0.91	<0.001
Infectious endocarditis: Any position IP, OT, or LT diagnosis during baseline year (Ref: No evidence)	0.22	1.25	0.97	1.62	0.087
Inhalant-related disorders: Any position IP, OT, or LT diagnosis during baseline year (Ref: No evidence)	0.99	2.69	1.95	3.71	<0.001
Lung disease: Any position IP diagnosis during baseline year (Ref: No evidence)	0.17	1.19	1.01	1.40	0.037
Lung disease: Any position OT or LT diagnosis during baseline year (Ref: No evidence)	0.12	1.13	1.01	1.26	0.040
Manic episode: Any position IP diagnosis during baseline year (Ref: No evidence)	-1.12	0.33	0.22	0.49	<0.001
Manic episode: Any position OT or LT diagnosis during baseline year (Ref: No evidence)	0.12	1.13	0.92	1.39	0.232
Nicotine: Any position IP diagnosis during baseline year (Ref: No evidence)	0.55	1.73	1.55	1.94	<0.001
Nicotine: Any position OT or LT diagnosis during baseline year (Ref: No evidence)	0.46	1.59	1.46	1.73	<0.001
Opioid use disorder: Any position OT or LT diagnosis during baseline year (Ref: No evidence)	1.76	5.81	5.34	6.32	<0.001
Opioid use disorder: Non-primary IP diagnosis during baseline year (Ref: No evidence)	1.80	6.03	5.27	6.89	<0.001
Opioid use disorder: Primary IP diagnosis during baseline year (Ref: No evidence)	2.29	9.83	8.11	11.92	<0.001
Other psychoactive substance: Any position IP diagnosis during baseline year (Ref: No evidence)	1.02	2.77	2.38	3.22	<0.001
Other psychoactive substance: Any position OT or LT diagnosis during baseline year (Ref: No evidence)	0.61	1.85	1.69	2.03	<0.001
Personality disorders Any position IP diagnosis during baseline year (Ref: No evidence)	0.24	1.27	1.01	1.59	0.040
Personality disorders Any position OT or LT diagnosis during baseline year (Ref: No evidence)	0.25	1.28	1.08	1.52	0.005
PTSD: Any position OT or LT diagnosis during baseline year (Ref: No evidence)	-0.07	0.93	0.82	1.05	0.252
Schizophrenia: Any position OT or LT diagnosis during baseline year (Ref: No evidence)	0.02	1.02	0.92	1.14	0.725
Schizophrenia: Non-primary IP diagnosis during baseline year (Ref: No evidence)	-0.54	0.58	0.47	0.73	<0.001
Schizophrenia: Primary IP diagnosis during baseline year (Ref: No evidence)	-0.28	0.76	0.60	0.96	0.023
Sedative/hypnotics/anxiolytic: Any position IP diagnosis during baseline year (Ref: No evidence)	-0.10	0.90	0.74	1.11	0.319
Sedative/hypnotics/anxiolytic: Any position OT or LT diagnosis during baseline year (Ref: No evidence)	0.28	1.33	1.12	1.56	<0.001

© 2025 Srivastava T et al. *JAMA Health Forum*.

Sexually transmitted infections: Any position IP diagnosis during baseline year (Ref: No evidence)	0.29	1.34	0.89	2.03	0.165
Sexually transmitted infections: Any position OT or LT diagnosis during baseline year (Ref: No evidence)	0.14	1.15	0.97	1.37	0.109
Sleep disorders: Any position IP diagnosis during baseline year (Ref: No evidence)	-0.59	0.56	0.45	0.68	<0.001
Sleep disorders: Any position OT or LT diagnosis during baseline year (Ref: No evidence)	-0.39	0.68	0.60	0.77	<0.001
Stimulants: Any position IP diagnosis during baseline year (Ref: No evidence)	1.35	3.87	3.39	4.42	<0.001
Stimulants: Any position OT or LT diagnosis during baseline year (Ref: No evidence)	1.25	3.48	3.14	3.84	<0.001

Notes:
- LASSO parameter estimates that show 0.00 are non-zero values that have been included in the model. 0.00 indicates that they're value is small enough to be rounded to 0.00 when using two digits after the decimal.
- Variables regarding pharmaceuticals (listed under *Individual-level clinical characteristics*) during the calendar year preceding the sampling year.
- α = 0.05
- Results for outcome D (Methamphetamines, ecstasy, or other psychostimulant-involved without opioid involvement) are reported in Table 2 in the manuscript's main text.

© 2025 Srivastava T et al. *JAMA Health Forum*.

eAppendix 6. Model fairness assessment with regards to sex and race/ethnicity variables.						
**Model Outcome (predicted risk ≥ 5%)**	**Full model**		**Model without Sex variable**		**Model without Race/Ethnicity variable**	
	**Sensitivity**	**PPV**	**Sensitivity**	**PPV**	**Sensitivity**	**PPV**
**A. Cocaine only with opioid involvement**						
Overall	1%	5%	1%	5%	1%	5%
Females	2%	100%	2%	100%	1%	100%
Males	1%	3%	1%	2%	1%	3%
White, non-Hispanic	1%	100%	1%	100%	1%	100%
Black, non-Hispanic	1%	1%	1%	1%	1%	2%
Hispanic, all races	1%	100%	1%	100%	1%	100%
**B. Cocaine only without opioid involvement**						
Overall	12%	7%	11%	7%	10%	7%
Females	8%	7%	10%	6%	7%	9%
Males	14%	7%	12%	9%	13%	7%
White, non-Hispanic	2%	2%	2%	2%	3%	2%
Black, non-Hispanic	17%	7%	17%	7%	14%	11%
Hispanic, all races	8%	10%	7%	9%	10%	3%
**C. Other stimulant with opioid involvement**						
Overall	1%	4%	1%	4%	1%	4%
Females	0%	100%	1%	100%	0%	100%
Males	2%	3%	1%	3%	2%	3%
White, non-Hispanic	1%	3%	1%	3%	1%	3%
Black, non-Hispanic					2%	100%
Hispanic, all races	1%	100%	1%	100%	1%	100%
**D. Other stimulant without opioid involvement**						
Overall	7%	6%	6%	5%	7%	6%
Females	3%	4%	4%	3%	3%	4%
Males	9%	6%	7%	7%	9%	6%
White, non-Hispanic	6%	5%	5%	4%	6%	5%
Black, non-Hispanic	10%	5%	9%	5%	11%	6%
Hispanic, all races	8%	7%	7%	7%	8%	7%

© 2025 Srivastava T et al. *JAMA Health Forum*.
