# Supplementary material for: Stimulant Overdose Prediction Model for Medicaid-Insured Persons
Source: JAMA Health Forum. 2025 Sep 19;6(9):e253489. doi: 10.1001/jamahealthforum.2025.3489 (PMC12449722; doi:10.1001/jamahealthforum.2025.3489)
Supplement: Supplement 2. — Data Sharing Statement [file jamahealthforum-e253489-s002.pdf]

Data Sharing Statement

Srivastava. Stimulant Overdose Prediction Model for Medicaid-Insured Persons. *JAMA Health Forum*. Published September 19, 2025. doi:10.1001/jamahealthforum.2025.3489

Data
Data available: No

Additional Information
Explanation for why data not available: Access to the raw, underlying Medicaid data used in this study is restricted due to patient privacy concerns.
